# Experimental autoimmune encephalomyelitis is associated with changes of the microbiota composition in the gastrointestinal tract

**DOI:** 10.1038/s41598-020-72197-y

**Published:** 2020-09-16

**Authors:** David M. Johanson, Jennifer E. Goertz, Ioana A. Marin, John Costello, Christopher C. Overall, Alban Gaultier

**Affiliations:** 1 Center for Brain Immunology and Glia, University of Virginia, Charlottesville, VA 22908 USA; 2 Department of Neuroscience, School of Medicine, University of Virginia, Charlottesville, VA 22908 USA; 3 Dept. of Neuroscience, Cornell University, Ithaca, NY 14850 USA; 4 Dept. of Neuroscience, Stanford University, Stanford, CA 94305 USA; 5 Myriad Genetics, Inc., San Francisco, CA 94080 USA

**Keywords:** Computational neuroscience, Data mining, Autoimmunity, Demyelinating diseases, Microbial communities

## Abstract

The gut microbiome is known to be sensitive to changes in the immune system, especially during autoimmune diseases such as Multiple Sclerosis (MS). Our study examines the changes to the gut microbiome that occur during experimental autoimmune encephalomyelitis (EAE), an animal model for MS. We collected fecal samples at key stages of EAE progression and quantified microbial abundances with 16S V3–V4 amplicon sequencing. Our analysis of the data suggests that the abundance of commensal Lactobacillaceae decreases during EAE while other commensal populations belonging to the Clostridiaceae, Ruminococcaceae, and Peptostreptococcaceae families expand. Community analysis with microbial co-occurrence networks points to these three expanding taxa as potential mediators of gut microbiome dysbiosis. We also employed PICRUSt2 to impute MetaCyc Enzyme Consortium (EC) pathway abundances from the original microbial abundance data. From this analysis, we found that a number of imputed EC pathways responsible for the production of immunomodulatory compounds appear to be enriched in mice undergoing EAE. Our analysis and interpretation of results provides a detailed picture of the changes to the gut microbiome that are occurring throughout the course of EAE disease progression and helps to evaluate EAE as a viable model for gut dysbiosis in MS patients.

## Introduction

Multiple Sclerosis (MS) is a prevalent autoimmune disease that leads to demyelination and neurodegeneration of the central nervous system (CNS)^1^. The main driver of injury in MS is the activation of T cells, which are thought to initiate myelin degradation and the development of autoimmunity^2^. T lymphocytes initially invade the CNS where they orchestrate the activation of CNS resident microglia and the recruitment of peripheral myeloid cells to attack and destroy myelin. Current therapies target the immune system and are associated with significant risk and incomplete efficacy, supporting the need for novel therapeutic approaches.

Although the etiology of MS is poorly understood, it is now clear that both genetic and environmental factors contribute to the disease^1^. Recent studies have begun to identify the gut microbiome as an active player in MS initiation and progression^3^. Indeed, the link between commensal microbiota and the immune system is becoming evident as the gut microbiota is essential for the education and maturation of immune cells and the immune system is key for surveillance and maintenance of a healthy gut flora^4^. It is well known that the gut microbiome is in a state of dysbiosis in both MS and experimental autoimmune encephalomyelitis (EAE), a well-accepted animal model of MS^3,5–7^. Multiple studies have shown that the microbiome is necessary to initiate disease, as germ-free (GF) animals^8^ or antibiotic-treated mice^9^ are resistant to EAE. Furthermore, Berer et al.^3^ utilized fecal samples obtained from monozygotic twin pairs discordant for MS to elegantly demonstrate that MS twin-derived microbiota induced a significantly higher incidence of EAE than the healthy twin-derived microbiota upon transfer in a GF spontaneous model of EAE^3^. Taken together, these pieces of evidence support a strong link between MS and the microbiome and warrant further studies to fully understand how the microbiome influences disease initiation and progression.

Here using EAE, we performed a longitudinal study to determine the microbiome composition at pre-onset, onset, peak and chronic phase with 16S-sequencing (16S-seq). Our study reveals a drastic reduction in *Lactobacillus* and an expansion of Clostridia bacteria, notably Peptostreptococcaceae. We applied multiple bioinformatics approaches to model how microbial populations and metagenome contents change over time. Our approach can be divided into three main arms: (1) Pairwise differential abundance analysis to reconstruct the changes in populations of individual bacterial taxa over time; (2) Construction of microbial co-occurrence networks to infer EAE-induced changes to microbe-microbe interactions and community structure from abundance data and; (3) Application of PICRUSt2 and weighted gene co-expression network analysis (WGCNA)^10^ to impute metagenomic abundances and identify putative associations between MetaCyc Enzyme Consortium (EC)^11^ pathways and disease parameters. Our study offers a detailed picture of gut microbiota dysbiosis over the course of EAE and benefits from a comprehensive dataset that spans multiple time points both before and during the disease. Our findings have the potential to generate hypotheses that could lead the way toward the development of gut centric therapeutic avenues for MS patients.

## Results

### EAE induced microbiome dysbiosis

**Figure 1:**
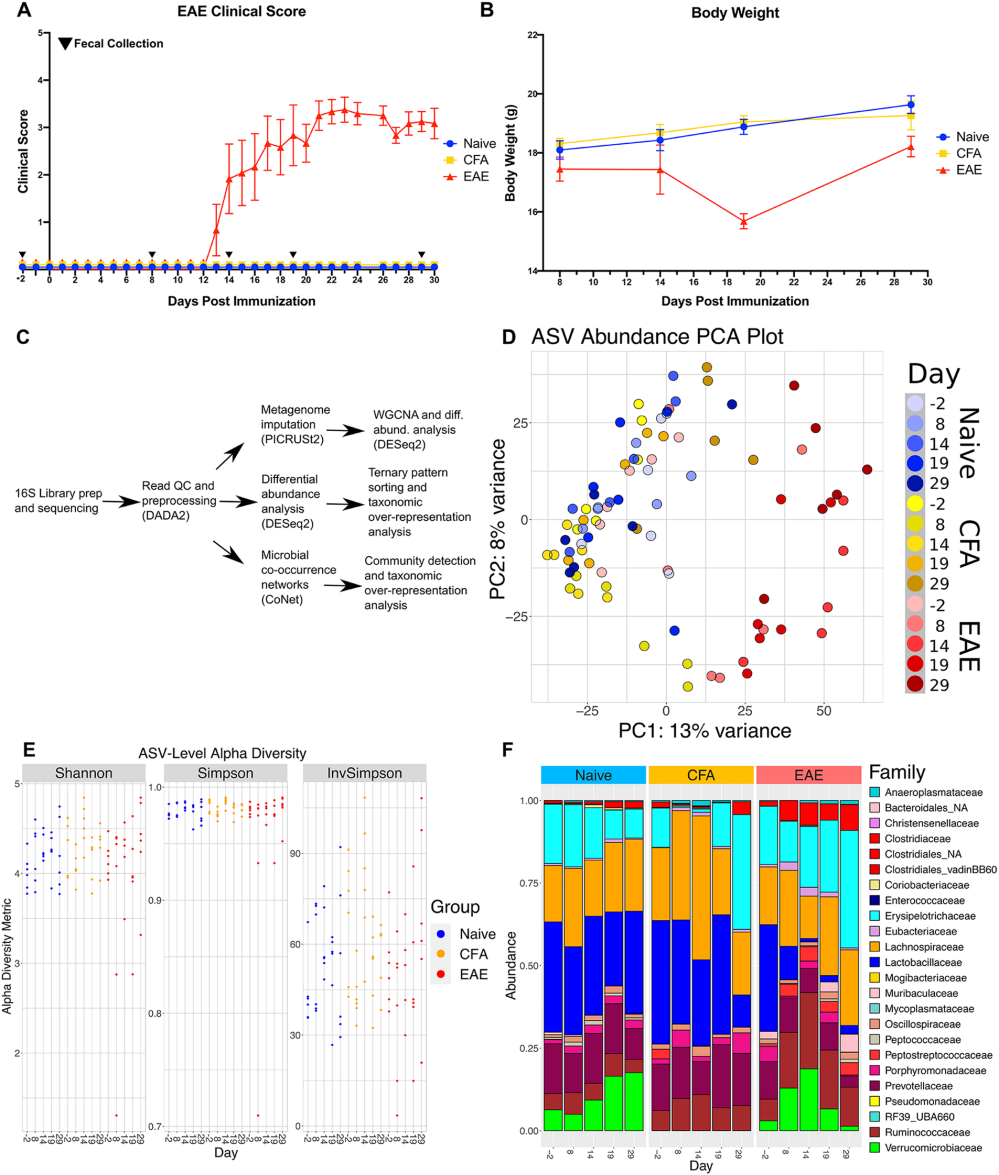
Microbiome dysbiosis is initiated following CFA and MOG immunization. (**A**) EAE clinical scores and (**B**) Weights of the 3 groups (Two-way ANOVA revealed that Time and Treatment accounted for 15.40% and 41.01% of variation respectively. P = 0.0121 and P < 0.0001 for each source). Black triangles represent days of fecal collection for 16S-seq libraries during course of experiment. (**C**) Workflow representing the bioinformatics applications and analyses applied to the 16S-seq dataset. The three main approaches include: differential abundance analysis, co-occurrence network analysis, and PICRUSt2 metagenome imputation. (**D**) PCA plot representing the first two principal components of the 16S ASV dataset after normalization and application of the variance-stabilizing transformation in DESeq2. Point color represents treatment group and days since immunization. (**E**) The Shannon, Simpson, and Inverse Simpson alpha diversity metrics for each sample plotted against dpi and colored by treatment group. Values were calculated with Phyloseq and are available in Table S3. (**F**) Stacked barplot of the relative abundances of each identified bacterial family. Relative abundances were derived from raw ASV abundances that had been grouped by family. Family bars are stacked in alphabetical order as they appear in the legend.

To study how EAE progression impacts the microbiota composition, cohorts of C57BL/6J mice were immunized with either an emulsion of myelin oligodendrocyte protein (MOG) and Complete Freund’s Adjuvant (CFA) (EAE group) or Complete Freund’s Adjuvant alone (CFA). Mice without immunization (naïve) were also included in our study. As expected, EAE group mice presented with ascending paralysis and weight loss that are characteristic of EAE (Fig. 1A,B), while the CFA and naïve groups appeared normal. Fecal pellets were sampled at different stages of the EAE (Fig. 1A) including before immunization (Day − 2), prior to symptom onset (Day 8), at early-onset (Day 14), at the peak (Day 19), and into the chronic phase of the model (Day 29). Next, we performed 16S-seq on fecal DNA isolated from naïve, CFA, and EAE animals at each time point (Fig. 1C). Principal component analysis (PCA) indicated that samples from EAE mice are distinct from the naive and CFA cohorts (Fig. 1D). The first principal component (PC1) separates EAE samples from CFA/naïve samples and can explain 13% of the variance among samples. The second principal component (PC2) accounts for 8% of total variance but does not appear to have a strong association with time or treatment. CFA samples at 8 dpi (days prior to or post-immunization), however, are separated from other CFA and naïve samples along PC2. PC1 is positively correlated with clinical score (r = 0.662, P-val = 2.101e−12, Table S2) and negatively correlated with weight (r = − 0.419, P-val = 2.962e−4, Table S2). PC1 visually segregates EAE samples from the rest of the dataset, and significant correlations with weight and score support the observation that EAE progression is associated with changes in gut microbiota. The distinction between EAE and other groups was also apparent in alpha diversity measurements (Fig. 1E). Indeed, examination of microbial diversity metrics calculated from Amplicon Sequence Variant (ASV) abundances revealed that alpha diversity decreases in EAE samples (Fig. 1E, Table S3). Shannon and Simpson metrics indicated that diversity is lower in EAE mice following immunization. The Inverse Simpson metric also indicates that diversity was lower but more variable among EAE mice following immunization (Fig. 1E).

At the family rank, the most drastic change was a decrease in Lactobacillaceae (blue) in the EAE group (Fig. 1F). Lactobacillaceae was the dominant family in naïve, EAE, and CFA mice before immunization, accounting for 33–37% of the gut microbiome (Table S1D). This observation holds true for the parent taxa, Bacilli and Lactobacillales, as well as the principal genus, *Lactobacillus*. The proportion of Lactobacillaceae started to decrease at EAE onset, 14 days after immunization (Figs. 1F, S1, Table S1D). Lactobacillaceae relative abundance also decreased in the post-immunization CFA group but not as robustly as the EAE group (Fig. 1F, Table S1D). Clostridia families (Clostridiaceae, Ruminococcaceae, Lachnospiraceae, and Peptostreptococcaceae) generally expanded in abundance after MOG immunization (Fig. 1F, Table S1D). More changes were observed at the genus rank; *Clostridium sensu stricto* *1*, *Ruminiclostridium*, *Ruminococcus*, *Romboutsia*, and *Corpococcus* presented with elevated abundance post-immunization (Fig. S1E, Table S1E). Another family, Erysipelotrichia maintained moderate abundance in naïve samples throughout the experiment, but was elevated in EAE immunized mice during the peak (day 19) and chronic (day 29) phases of the disease (Fig. 1F, Table S1D). Taken together our data suggest that EAE induces a rapid dysbiosis characterized by *Lactobacillus* depletion and dramatic growth in *Romboutsia* and various Clostridia and Ruminococcaceae populations. Surprisingly, immunization with CFA alone was sufficient to drive similar change in microbiota composition albeit to a lesser extent.

### Differential abundance analysis highlights a subset of EAE-responsive microbes

To characterize the response profile of every detectable taxon in our dataset, we applied DESeq2, a computational tool to perform differential abundance analysis^12^. Differential abundance analysis was applied in two major frameworks. The first involves pairwise comparisons between two experimental groups at each time point (i.e. pairwise comparisons at − 2, 8, 14, 19, and 29 dpi). This framework was applied to each cross-treatment pair independently (EAE/naïve, CFA/naïve, and CFA/EAE). The second testing framework focused on comparing every time point within a single treatment group to the corresponding pre-immunization time point (i.e. CFA 8/− 2, CFA 14/− 2, CFA 19/− 2, and CFA 29/− 2). This framework was performed on naïve, EAE, and CFA samples. The analysis was independently repeated on abundance data that had been grouped (agglomerated) at each taxonomic rank (phylum, class, order, family, genus, species, ASV) to obtain differential abundance results for all taxa.

**Figure 2:**
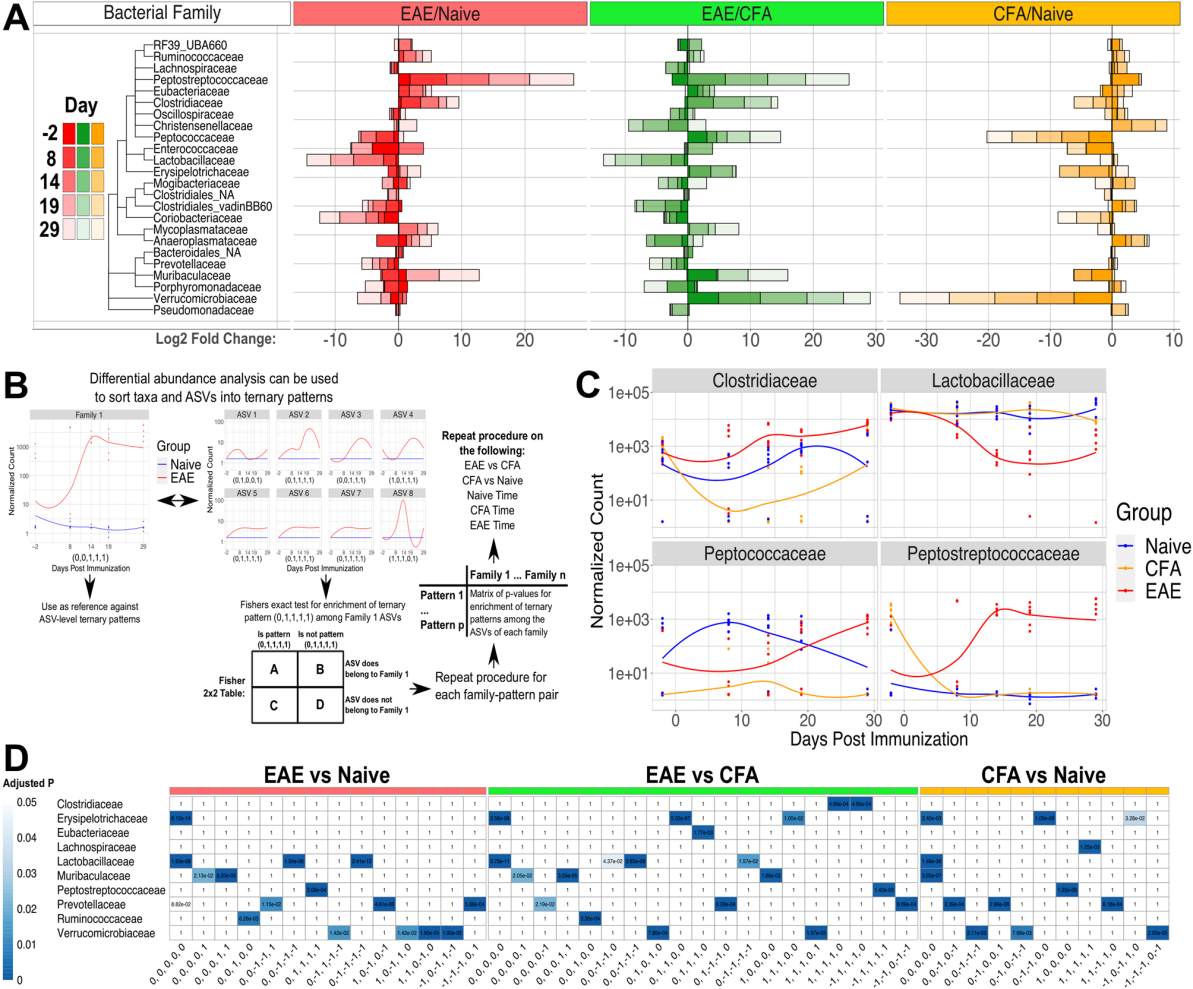
Differential abundance analysis reveals key taxa changes. (**A**) Stacked barplot of DESeq2 log2 fold change values for pairwise comparisons across experimental group. Each bar represents an individual pairwise comparison between two experimental groups made at a specific time point. Tree represents approximate taxonomy of the bacterial families and was determined by manual supplementation of NCBI taxonomy. (**B**) Schematic illustrating the process of creating ternary patterns and performing taxonomic over-representation analysis on each ternary pattern and taxa pairing. Differential abundance analysis was performed at specified taxonomic levels allowing for the identification of ASVs with different patterns from their parental taxa. Fisher’s exact test for enrichment of ternary patterns among the ASVs of a given taxon was applied. Results were compared to the ternary pattern derived from the entire taxon as well, yielding new insight. This testing framework was applied independently for each ternary pattern. (**C**) Normalized abundances for four bacterial families of interest that were identified from differential abundance analysis results or taxonomic over-representation analysis results. Dots represent normalized count abundances of individual samples and are plotted against time. (**D**) Heatmap of taxonomic over-representation analysis results. Color is scaled from 0 to 0.05 and represents the BH-adjusted P-value derived from Fisher’s exact test for enrichment of ternary pattern among a taxon.

Lactobacillaceae and Peptostreptococcaceae exhibited opposite patterns during the course of EAE disease progression (Fig. 2A, Tables S5, S7D). Lactobacillaceae was significantly depleted at all time points following symptom onset (14, 19, and 29 dpi) in EAE/naïve comparisons (Fig. 2A: log2 fold change (L2FC) = − 5.329, − 3.653, − 4.072, P-adj. = 1.92e−13, 2.33e−7, 6.6e−9, Table S5A–E). Within-treatment differential abundance analysis also suggests that Lactobacillaceae was depleted in EAE mice after the pre-clinical phase (8 dpi) (14/− 2, 19/− 2, 29/− 2 dpi: L2FC = − 5.223, − 3.926, − 3.161, P-adj. = 1.25e−12, 8.38e−8, 1.96e−5, Table S5X-AA). Conversely, Peptostreptococcaceae was elevated in EAE/naïve comparisons following immunization (Fig. 2A: 8, 14, 19, 29 dpi: L2FC = 9.567, 10.046, 10.355, 10.581, P-adj. = 3.02e−14, 1.09e−14, 9.4e−17, 2.26e−17, Table S5A–E). EAE/CFA comparisons supported this observation (Fig. 2A, Table S5F–J). Bacterial genera *Romboutsia* and *Lactobacillus* exhibited the same differential abundance patterns as their parent families, Peptostreptococcaceae and Lactobacillaceae (Figs. S2E, S3E, Table S5A–E). Mycoplasmataceae, Clostridiaceae and Ruminococcaceae were elevated in EAE/naïve comparisons, but Clostridia from the vadinBB60 group (Clostridiales_vadinBB60) were depleted in abundance from 8 dpi onward (Fig. 2A, Table S5A–E). Bacterial genera including *Mycoplasma*, *Muribaculum*, *Ruminiclostridium*, *Massilioclostridium*, *Tyzzerella, Anaerotignum, and Marvinbryantia* were also impacted by EAE as seen in the EAE/naïve and EAE/CFA comparisons (Figs. S2E, S3E, Table S5A–J). None of the aforementioned taxa were differentially abundant on -2 dpi in EAE/naïve or EAE/CFA comparisons (Figs. S2E, S3E, Table S5A–J), suggesting that these results were triggered by EAE.

### Over-representation analysis of bacterial taxonomy and temporal trajectory reveals that bacterial ASVs take trajectories largely representative of parent taxa

Bacterial taxonomy is an imperfect classification strategy; species and subspecies within a genus can have different metabolic proficiencies, occupy different niches, or even directly compete with each other with varying levels of fitness^13–16^. Because of this, we applied Fisher’s exact test for over-representation of ternary patterns within bacterial taxa to examine subpopulations of ASVs in each taxa that exhibit heterogeneous responses to immunization with MOG and CFA (Fig. 2B). This approach is also known as over-representation analysis (ORA). The rationale for this approach was partially inspired by established applications of Fisher’s exact test with respect to gene ontology^17^. The same six differential abundance analysis frameworks were applied on un-agglomerated ASVs data to create ternary patterns (Tables S4, S7). Each pattern consists of a sequence of digits representing the differential abundance result at a given time point (1 represents an increase in abundance (L2FC > 0, P-adj. < 0.05), − 1 a decrease in abundance (L2FC < 0, P-adj. < 0.05), and 0 an instance of no significant change (P-adj. > 0.05)). These ternary patterns are generally faithful to the normalized abundances of the taxa that they are calculated from (Fig. 2C, Table S7). Fishers exact test was applied to test for over-representation of a ternary pattern among the ASVs of a taxon. This was re-applied independently at each taxonomic rank and on all six testing frameworks. The background distribution included all 2,295 ASVs.

We found that bacterial phyla, classes, and orders possess significant associations with multiple ternary patterns, regardless of the pairwise or within-treatment comparison being made (Figs. S4A–C, S5A–C, Table S6A–F). It is unsurprising that we find multiple taxon-pattern associations when Fisher’s exact test is applied at higher taxonomic ranks, given that these are broad labels with diverse constituents. At the family rank, Lactobacillaceae ASVs were associated with three distinct ternary patterns in the EAE/naïve framework (“0,0,0,0,0”, “0,0,− 1,− 1,− 1”, and “0,− 1,− 1,− 1,− 1”; P-adj: 1.93e−8, 1.54e−6, 2.41e−12, Fig. 2D, Fig. S4D, Table S6A). These results imply that not all Lactobacillaceae ASVs respond to EAE and also suggests the existence of population subsets that respond to EAE at different stages. Examination of ORA results at the species rank reveals that ASVs belonging to *Lactobacillus johnsonii* exhibited associations with ternary patterns indicative of depleted abundance during EAE, whereas ASVs belonging to *Lactobacillus johnsonii/tiawanesis* (ambiguous taxonomy) were significantly associated with the “0,0,0,0,0” pattern of no change during EAE (Fig. S4F, Table S6A). In EAE/CFA comparisons, we found significant enrichment of Lactobacillaceae ASVs in an additional fourth pattern (“0,0,− 1,− 1,0”, “0,0,0,0,0”, “0,0,− 1,− 1,− 1”, and “0,− 1,− 1,− 1,− 1”: P-adj : 3.75e−11, 2.85e−8, 1.57e−2, 4.37e−2, Fig. S4D, Table S6B). In contrast, Peptostreptococcaceae was enriched in a single EAE/naïve pattern “0,1,1,1,1” and the EAE/CFA pattern “− 1,1,1,1,1” (P-adj: 3.09e−4, 1.42e−3, Fig. S4D, Table S6A,B). Other taxa of interest included Prevotellaceae and Muribaculaceae (Fig. S4D, Table S6). Some of these taxa were enriched in ASVs following the CFA/naïve “0,0,0,0,0” ternary pattern, or another ternary pattern that suggests that CFA alone does not account for the observed differential abundance patterns observed in EAE/naïve comparisons. Fishers exact testing on ternary patterns largely agrees with the observations made from the ternary patterns derived from agglomerated abundance data (Fig. 2A,D, Figs. S2, S3, S4, S5, Tables S6, S7). These results reinforce observations made from differential abundance testing on agglomerated taxa and inform them by suggesting that further subdivisions can be made within these taxa to identify subspecies or strains that drive observed responses to EAE and CFA immunization.

### Lactobacillus reuteri dietary supplement alters EAE progression

**Figure 3:**
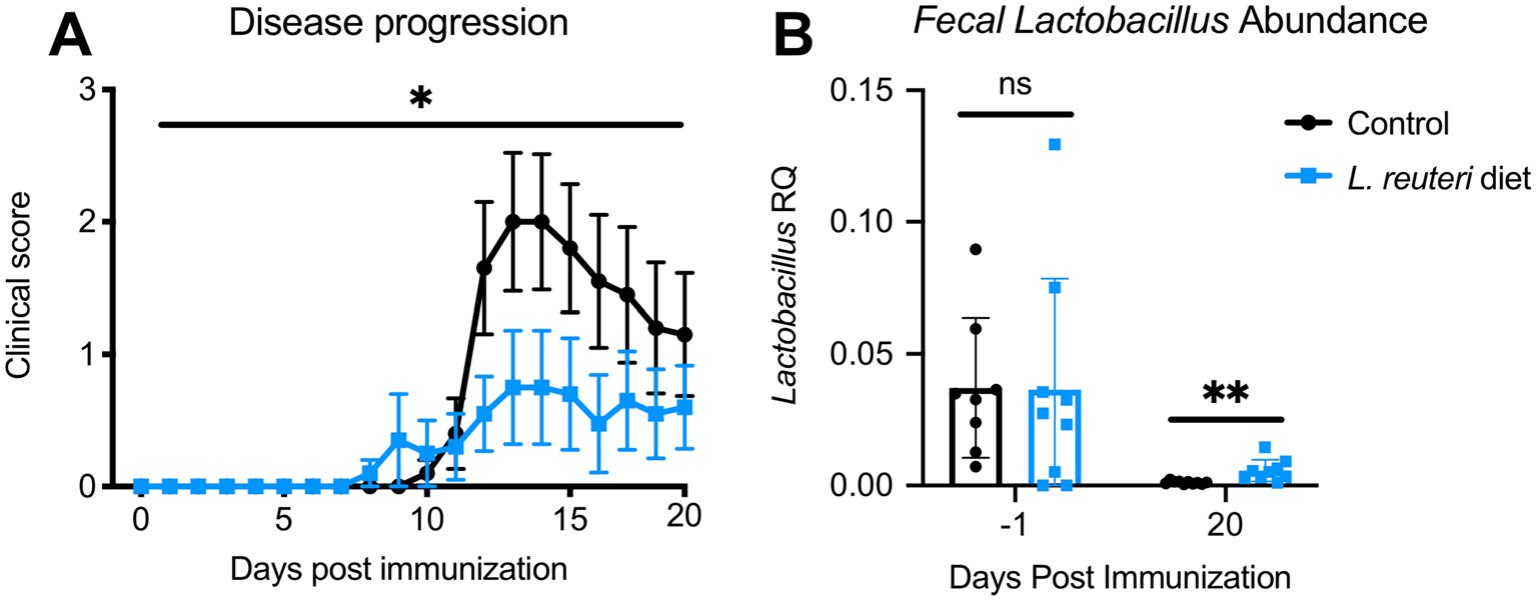
Treatment with *L. reuteri* is protective in EAE. (**A**) Mice fed with *L. reuteri* present with lower clinical scores than Broth control (Wilcoxon matched-pairs signed-rank test: p-value = 0.0132, n = 10 per group). (**B**) qPCR with *Lactobacillus* specific primers revealed that abundance of *L. reuteri* is elevated in mice fed with supplemented diet 20 dpi (Paired t-tests at − 1 and 20 dpi: P-values = 0.9716 and 0.0054, n = 10 per group).

Our data show that *Lactobacillus* is significantly decreased in the course of EAE and could be a keystone species based on our microbiota community analysis (Figs. 1 and 2). Given that important function, we wanted to assess the role of *Lactobacillus* on EAE. Mice were immunized with MOG_35-55_ and provided with daily feed that had been mixed with a *Lactobacillus Reuteri* overnight culture or sterile broth control as previously described^18^. Both experimental groups presented ascending paralysis and weight loss, but *Lactobacillus*-enriched mice presented with a significantly reduced average clinical score when compared to control mice (Fig. 3A). Our study confirms previous work demonstrating that administration of *Lactobacillus Reuteri* is protective in EAE^19^. Fecal pellets were sampled before immunization (− 1 dpi) and at 20 dpi. As expected, qPCR analysis revealed that *Lactobacillus* 16S V3–V4 amplicon abundance was higher in the stool of *Lactobacillus*-enriched mice than control mice (Fig. 3B).

### Co-occurrence and mutual exclusion network analysis reveals changes in the microbiome “interactome” due to EAE or CFA immunization

**Figure 4:**
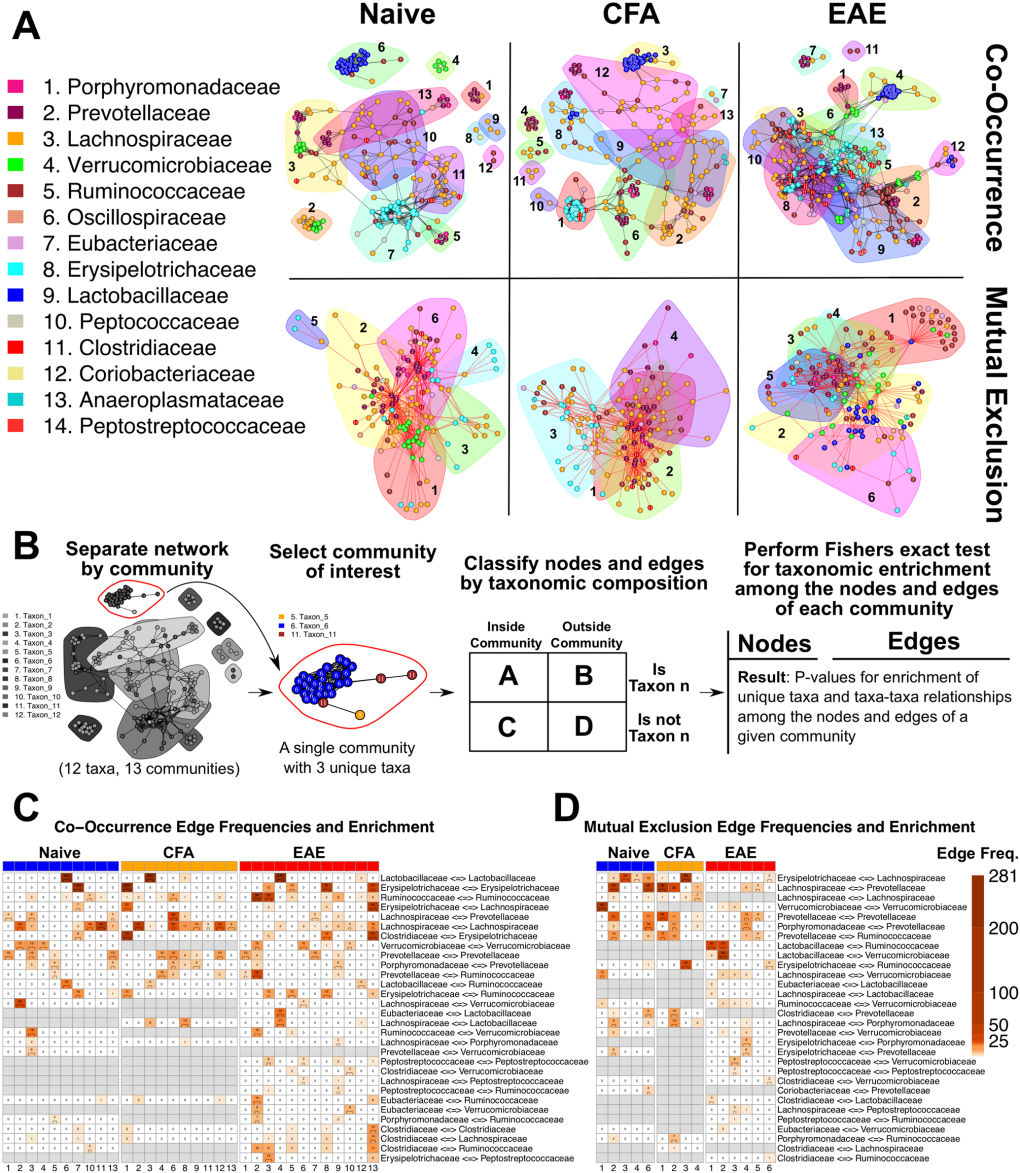
Co-occurrence network analysis with the ReBoot algorithm reveals novel community interactions in EAE. (**A**) Color-coded network graph representations of the co-occurrence and mutual exclusion interactions among ASVs. White numbers within nodes correspond to numbering in the legend. Transparent shapes represent network communities determined by the Louvain modularity algorithm. Black numbering corresponds to the numbering given to distinguish communities within each network. (**B**) Schematic representing the process for calculating taxonomic over-representation with respect to the nodes and edges within a community. Networks are split by community membership and tested for over-representation of all taxa and all unique taxa-taxa pairings within the community of interest. Fisher’s exact test is applied on two-way contingency tables that distinguish nodes by community membership and taxonomic membership. Testing framework is performed independently for each network. (**C**) Heatmap of BH-adjusted P-values for over-representation of unique edges among the communities of each co-occurrence network. Each row represents a unique edge connecting two distinct families. Each column is a community in one of the three networks. Cell color corresponds to edge frequency within each network and each cell contains an edge frequency number followed by a significance level in parentheses (Blank = n.s., *P ≤ 0.05, **P ≤ 0.01, ***P ≤ 0.001, ****P ≤ 0.0001). Heatmap has been filtered to only contain edges of interest and communities with at least one significant P-value among these edges. Grey cells represent edges that were entirely absent from the network they appear under. Columns represent unique communities in each network. (**D**) Heatmap representing BH-adjusted P-values for over-representation of unique edges among the communities of each mutual exclusion network following the same construction methods and conventions as (**B**). Columns represent unique communities in each network. The manually selected edges of interest are different from those in (**C**).

We next wanted to explore microbe–microbe interactions among ASVs to understand changes that occur to microbial community organization during EAE and to find potential mediators of dysbiosis. This was accomplished by creating microbial co-occurrence and mutual exclusion networks with the ReBoot algorithm on the Cytoscape “CoNet” application^20,21^. The 16S abundance dataset was split by experimental group to create three independent networks. In co-occurrence networks, nodes are used to represent microbes (in this case ASVs) and edges represent microbe–microbe interactions. Each network was split into two network subgraphs to segregate the positive (co-occurrence) edges from the negative (mutual exclusion) edges to make interpretation more straightforward (Fig. 4A,B). Networks were partitioned into communities using the Louvain algorithm to identify centers of increased modularity (a measure of network organization). Community significance was determined with the Wilcoxon Rank-Sum test (Table S12A–F).

The final networks indicated a general interaction structure that changes in response to EAE or CFA immunization. Fisher’s exact test was applied to find evidence for taxonomic over-representation within each module against the network as a whole. This testing framework was also applied to unique edges (Fig. 4B). Many community structures are conserved across the naïve, CFA, and EAE networks. When the Jaccard Index was applied to identify communities that share identical ASVs among networks, specific ASVs appeared in multiple networks (Fig. S6). This is most evident among co-occurrence networks, where each network-network pairing contains at least two community pairs with indices between 0.5 and 0.8. An inspection of edge and node counts and the over-representation analyses results also support this conclusion. All three co-occurrence networks contain at least one large community that is enriched in Lactobacillaceae (naïve 6, CFA 3, EAE 4: P-adj: 6.163e−28, 2.269e−26, 6.880e−22, Fig. 4C, Fig. S9D, Table S9D), but the CFA and EAE co-occurrence networks contain additional Lactobacillaceae ASVs in other communities (Fig. 4A, Fig. S9D, Table S9D, S11). Only the EAE mutual exclusion network contained Lactobacillaceae ASVs; it also possessed a community with a significant enrichment in Lactobacillaceae (EAE 2: P-adj: 6.665e−8; Fig. 4D, Fig. S9D, Table S9D).

Lactobacillaceae are largely isolated from other taxa in the naïve co-occurrence network; only a few Ruminococcaceae ASVs share edges with Lactobacillaceae (Fig. 4A, Fig. S8D, Table S10D). This is not so in the CFA co-occurrence network, where Lactobacillaceae ASVs share edges with Ruminococcaceae and Lachnospiraceae ASVs (Fig. 4A, Fig. S8D, Table S10D). Lactobacillaceae ASVs in the EAE networks share edges with ASVs from the Eubacteriaceae, Lachnospiraceae, Oscillospiraceae, Prevotellaceae, Ruminococcaceae, and Verrucomicrobiaceae families (Fig. S8D, Table S8D, S10D). Both the CFA and EAE co-occurrence networks also possess one community with enrichment in Lachnospiraceae⇔Lactobacillaceae edges (CFA 8: n = 14, P-adj = 3.31e−12, EAE 4: n = 17, P-adj = 4.91e−9, Fig. 4C, Fig. S8D, Table S8) and enrichment in Lactobacillaceae⇔Ruminococcaceae edges (CFA 3: n = 6, P-adj = 7.85e−3, EAE 12: n = 6, P-adj = 5.97e−12, Fig. 4C, Table S8D). The EAE group also has a unique community with enrichment for Eubacteriaceae⇔Lactobacillaceae edges (EAE 4: n = 19, P-adj = 1.91e−11; Fig. 4C, Fig. S8D, Table S8). Lactobacillaceae in the EAE mutual exclusion network shares edges with Clostridiaceae, Eubacteriaceae, Lachnospiraceae, Prevotellaceae, Ruminococcaceae, Verrucomicrobiaceae, and Peptostreptococcaceae ASVs (Fig. S8D, Table S8D, S10D). Peptostreptococcaceae nodes are exclusive to EAE networks (Fig. 4A,C,D, Fig. S9D, Table S9D, S11D) and they share edges with nodes belonging to 10 other families. Lachnospiraceae, and Verrucomicrobiaceae also exhibit altered structure among communities in EAE networks. These taxa are organized into homogenous groups of nodes in naïve and CFA networks, but they share more edges with ASVs from other families than each other in EAE networks (Fig. 4A, Fig. S8D, Table S10D). The new interactions between Lactobacillaceae and other families in both EAE networks may be reflective of an altered gut microbial ecology during EAE.

Our network analysis expands upon the theme of Clostridia expansion during EAE that was observed in relative abundances (Fig. 1F) and differential abundance testing (Fig. 2A) by suggesting that this “EAE-induced Clostridia expansion” disturbs the pre-existing community structure of the gut microbiome. We observed new Clostridia nodes, the disruption of naïve edges by Clostridia edges, and novel taxa in CFA and EAE networks. The networks implicate Ruminococcaceae and Lachnospiraceae—among others—as being important mediators of the global “interactome” architecture and suggest that EAE alters their normal interactions with other taxa. Lactobacillaceae may be competing in an altered microbial ecosystem following immunization with MOG that is preventing its return to pre-immunization abundances. Taken together, these co-occurrence and mutual exclusion networks help visualize a simplified topology of the “interactome” and highlight specific changes that may be occurring during EAE. Network modules were also subject to PCA. Correlation coefficients between phenotypic traits and the primary principal components of each module are available in Fig. S10. Network visualizations at each taxonomic level can be found in Fig. S7.

### Metagenome imputation with PICRUSt2 identifies MetaCyc Enzyme Consortium pathways and accessions that are associated with EAE disease progression and weight loss

**Figure 5:**
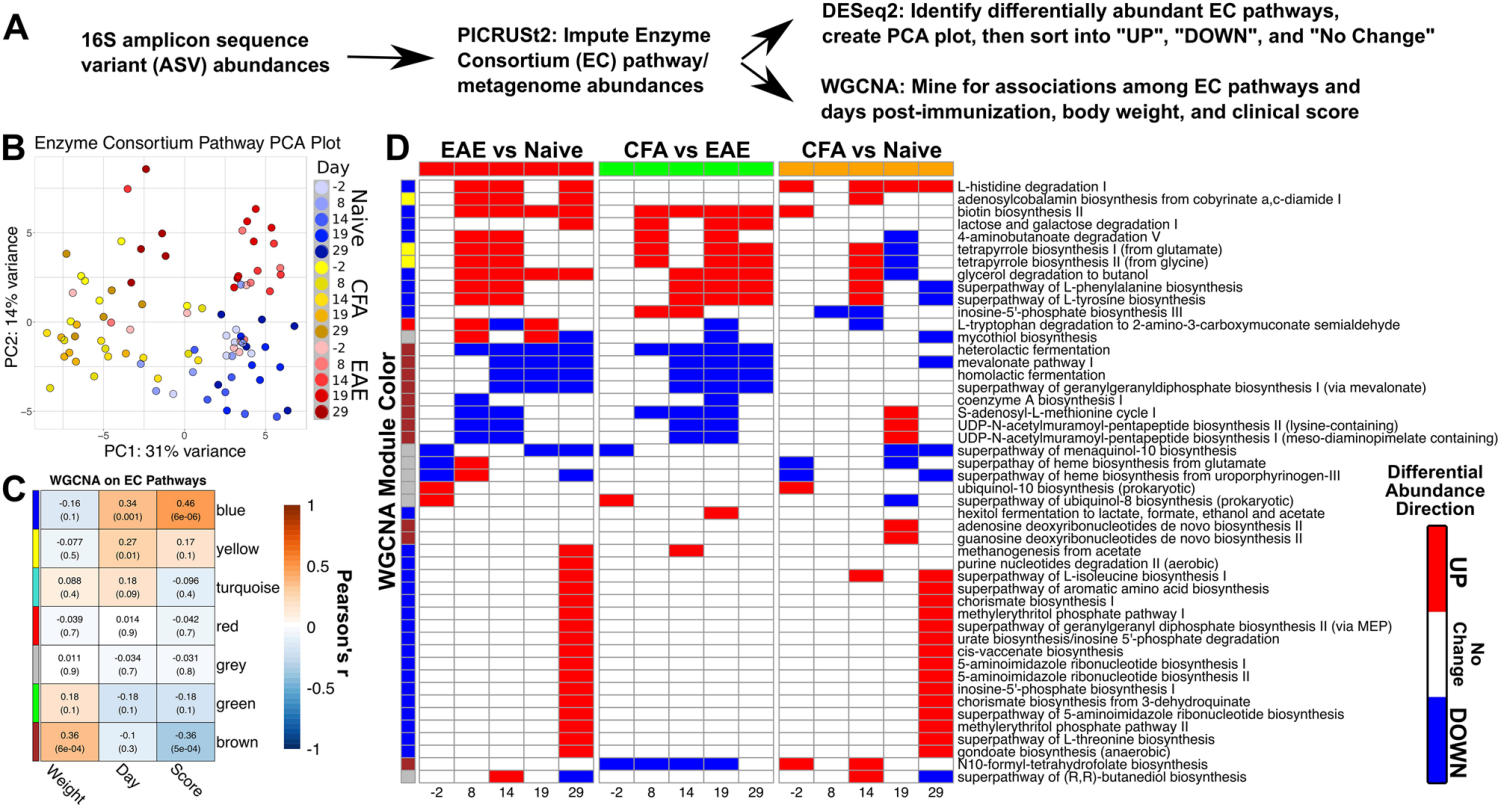
Abundance derived metagenomics data highlight putative EAE induced metabolic changes. (**A**) Investigation of PICRUSt2-imputed data involved applications of WGCNA and DESeq2 differential abundance analysis to identify groups of MetaCyc EC accessions and pathways potentially associated with EAE. EC pathways and EC accessions were analyzed independently. (**B**) PCA plot of first two principal components of EC pathway data. Raw output from PICRUST2 was normalized and subject to a variance-stabilizing transformation within DESeq2. Samples from each groups diverge as dpi increases. (**C**) Heatmap of modules derived from WGCNA on EC pathways. Cell color represents Pearson’s r between the first principal component of each module and clinical score, dpi, and body weight. Vertical color bar represents the color for each WGCNA module. Cells contain the rounded Pearson’s r and P-values in parentheses. (**D**) Curated heatmap of log2 fold changes of DESeq2 differential abundance analysis when applied to the EC pathway abundances. Cell color represents the directionality and significance of the log2 fold change vales for each pairwise comparison. Red represents significant increase (P ≤ 0.05, L2FC > 0), blue represents significant decrease (P ≤ 0.05, L2FC < 0), and white represents no significant change (P > 0.05). Each row represents an EC pathway of interest and column numbering represents the dpi that a pairwise comparison was made. Vertical color bar conforms to the same convention as (**C**) and denotes the module membership of each EC pathway.

One way the gut microbiome can influence host physiology is through the generation of soluble metabolites that can cross the intestinal mucosa and enter circulation^22^. PICRUSt2 was used to impute the metagenomic contents of the gut microbiota from 16S-seq abundances^23–27^ (Fig. 5A). The imputed data included abundances for EC accessions and corresponding EC pathways. DESeq2 was applied to normalize imputed EC pathway and accession abundances with respect to the same experimental model used on ASV abundances (~ Treatment + Time). Normalized EC abundances were then subject to weighted gene correlation network analysis (WGCNA)^10^ to find modules of EC pathways or accessions (Figs. S11, S13, Tables S13B, S14B, S16) that possess significant correlations with weight, clinical score, or day. PCA on EC pathway and accession data was able to resolve experimental groups to a greater degree than the PCA on normalized 16S amplicon abundances (Fig. 5B). 47% of the total variance among EC pathway samples was represented by the first two principal components (Fig. 5B). PC2 was positively correlated with clinical score (r = 0.574, P-val = 4.986e−9, Table S14A) and negatively correlated with weight (r = − 0.430, P-val = 1.998e−4, Table S14A). The cumulative variance contained in PC1 and PC2 rises to 57% when PCA was applied to DESeq2-normailzed EC accession data (Fig. S11A).

WGCNA on EC Pathways produced two modules of interest that had significant correlation coefficients with clinical score. The brown module was negatively associated with score (r = − 0.36, P-val = 5e−4, Fig. 5C) and positively associated with weight (r = 0.36, P-val = 6e−4, Fig. 5C). The blue module was positively associated with score (r = 0.46, P-val = 6e−6, Fig. 5C) but has no significant association with weight (r = − 0.16, P-val = 0.1, Fig. 5C). While these modules as a whole exhibited certain associations with score, weight, and day, some EC pathways differed from module-level observations. All EC pathways from the brown module were negatively correlated with day and score, but positively correlated with weight. EC pathways from the brown module that decreased in EAE included heterolactic fermentation, homolactic fermentation, coenzyme A biosynthesis, and the *S*-adenosyl-l-methionine cycle (Fig. 5D, Fig. S12, Table S15). Blue module EC pathways overwhelmingly had positive correlations with score and dpi, but some pathways possessed negative correlations with weight (Table S13A). Blue module pathways were involved with degrading l-Histidine, 4-aminobutanoate, lactose, and galactose (Fig. 5D, Table S13A). The blue module also contains EC pathways responsible for the synthesis of butanol, l-phenylalanine, l-tyrosine, inosine-5′-phosphate, 5-aminoimidazole, l-threonine, and gondoate and implicates them in being associated with EAE clinical score. Differential abundance testing supports this association (Fig. 5D, Table S15). Most EC pathways associated with the blue module experienced an increase in abundance in EAE mice relative to naïve mice by the chronic phase of disease at 29 dpi. All EC pathways from the brown module were present in significantly lower abundances in EAE mice relative to naïve and CFA mice. However, some of these pathways are elevated in naïve/CFA comparisons at 29 dpi. No EC terms from the blue or brown modules exhibit differential abundance when naïve or CFA mice are compared against EAE pathways prior to immunization (− 2 dpi). This is important given that all mice are naïve at this time in the experiment. These results come from imputed data and cannot replace experimental verification, but they could help identify pathways or microbiome targets with a therapeutic potential for patients with MS and a number of other human conditions.

## Discussion

Multiple sclerosis (MS) is a common neurodegenerative disease in which the central nervous system myelin is destroyed by the immune system. Many studies have described the presence of microbiota dysbiosis in MS patients but the precise role of the microbiome in MS pathogenesis and progression remains unclear^3,5,7^. Human microbial studies on MS are complicated by factors such as genetic make-up and geographical and environmental variations such as disease modifying therapy. Studies performed with the animal model of MS, EAE, have also shown evidence of disrupted microbiota dysbiosis but have, to date, only included end-point microbiome analysis, preventing the exploration of changes to microbiome composition during the full course of the disease model^6^. Here, we performed a longitudinal analysis of the gut flora at pre-onset, onset, peak, and chronic phase of EAE to obtain a comprehensive dataset of the microbiome changes associated with EAE. Our study design also included a cohort of animals injected with CFA to decouple the effect of immune system activation from the autoimmune responses of EAE on gut flora.

Our study demonstrates that one of the most robust changes at the family rank was a decrease in Lactobacillaceae starting at EAE onset. This observation was conserved for the parent taxa Bacilli and Lactobacillales, and in the genus, *Lactobacillus*. Commensal *Lactobacillus* is a critical component of the healthy gut flora as they present with protective functions against infection, such as the production of anti-microbial agents^28,29^. Lactobacillus dysbiosis was also shown to be present in an animal model of depression and therapeutic supplementation with *L. reuteri* was sufficient to improve depressive-like symptoms^18^. Here, we have demonstrated that *L. reuteri* supplementation was beneficial in mice undergoing EAE, further confirming a recent study^19^. Our community analysis suggests that *Lactobacillus* may be a keystone species of the gut flora, as its decrease correlates with microbial community reorganization. Taken together these results point towards further exploration of *Lactobacillus* supplements as a potential treatment for MS and other conditions associated with gut dysbiosis^19^. It remains unclear which factors drive the decrease in *Lactobacillus* levels, but our results obtained from mice immunized with CFA alone also indicate some reduction in *Lactobacillus*, perhaps suggesting that the immune system might be involved. Further work is warranted to determine the factor(s) impacting *Lactobacillus* abundance in the gut. Furthermore, given the growing evidence supporting the effect of *Lactobacillus* as a regulator if the immune system, it is also critical to determine its mechanism of action in the context of EAE^30,31^.

As Lactobacillaceae levels decline, members of the Clostridia families (Clostridiaceae, Ruminococcaceae, Lachnospiraceae, and Peptostreptococcaceae) and the Erysipelotrichia family expand. Expansion of Clostridia has been reported in DMT naïve MS patients^32,33^, but the contribution of Clostridia to MS pathogenesis remains unclear as transfer of Clostridia isolated from MS patients to mice treated with antibiotics did not alter the course of EAE^32^. Taken together, our data suggest that EAE induces a reorganization of the gut flora characterized by *Lactobacillus* depletion and expansion of Clostridia populations; most notably Ruminococcaceae and Peptostreptococcaceae. Future sequencing studies aimed at exploring these differences in various regions of the intestine (eg: small intestine vs colon) are critical to demonstrate the main location(s) of these EAE induced changes. While our study was unable to establish a causative link between Clostridia and dysbiosis, it did identify a number of species for further investigation.

Our application of PICRUSt2 to impute metagenomic data from 16S-seq abundances was intended to explore links between the host and the gut microbiome in the context of EAE. As an example, our data suggests that the mevalonate and menaquinol-10 pathways are significantly decreased in EAE when compared to naïve and CFA mice. These pathways are essential for the generation of vitamin K_2_ by bacteria. Vitamin K_2_ is important for numerous host functions including blood coagulation^34^ and bone homeostasis^35^. Recent research has demonstrated that MS patients have depleted Vitamin K_2_ levels when compared to controls^36^. Interestingly, Vitamin K_2_ has been shown to block the proliferation of human activated T lymphocytes^37^, a hallmark event of MS pathology and EAE^38^. In sum, our data supports observations that Vitamin K_2_ supplementation alters the progression of MS in patients and suggests specific bacterial pathways related to the role of Vitamin K_2_ in MS to be further investigated as potential therapeutic targets.

Metagenome imputation and differential abundance analysis also suggests that genes responsible for 4-aminobutanoate (GABA) degradation were more abundant in the EAE cohort. Recent work has started to show that the ability of the gut flora to produce and degrade neurotransmitters, such as GABA could have significant impact on the host nervous system^39^. In addition to its function as an inhibitory neurotransmitter, GABA has been shown to inhibit T cell function^40,41^. GABA can block the immune response following bacterial infection or in the context of autoimmunity^40,41^. Since GABA can be produced by many gut resident bacteria, including *Lactobacillus*^42,43^, we postulate that increased GABA degradation in the intestine could lead to an unshackled immune response. Furthermore, GABA can act as a quorum signaling molecule controlling biofilm production^44,45^. Further investigation is needed to determine the effects that bacterial GABA flux exerts on the community structure of the gut microbiome and the overall host immune state. More specifically, investigation into the roles that bacterial and host GABA play in the context of EAE is needed. In sum, our longitudinal microbiome sequencing study has revealed some dynamic changes to the gut flora during the course of EAE disease progression. Our analysis also highlights, for the first time, the impact of CFA on gut microbial composition. Our dataset could give rise to new hypotheses that may lead to the development of novel therapeutic approaches to MS that target the gut microbiome or its metabolic output. We have also prepared a detailed supplementary section with analysis results for each taxonomic rank included in this study.

## Methods and materials

All methods were carried out in accordance with relevant guidelines and regulations.

### Experimental autoimmune encephalomyelitis

Female C57BL/6J mice (8 weeks old) were purchased from the Jackson Laboratory. EAE was induced and scored as previously described^46^. Briefly, female C57BL/6 mice were immunized subcutaneously with 50 μg of myelin oligodendrocyte glycoprotein peptide 35–55 (MOG_35–55_) (CSBIO #CS0681) emulsified at a 1:1 ratio in Complete Freud’s Adjuvant (Sigma Aldrich #F5881). On days 0 and 2, 250 ng of pertussis toxin (List Biologicals #180) was administered intraperitoneally. For EAE experiments involving dietary supplementation, *L*. *reuteri* was purchased from ATCC (#23272) and cultured aerobically according to the manufacturer. Mice were fed ad libitum starting the day after immunization with pulverized food pellets mixed with fresh *L. reuteri* or MRS culture broth as described^18^. All animal experiments were approved and complied with regulations of the Institutional Animal Care and Use Committee at University of Virginia (#3918).

### 16S read collection and quantification

Fecal Samples were collected from mice at two days prior to EAE and CFA group immunizations and at 8, 14, 19, and 29 days after immunization. Fecal samples were then processed for 16S-seq and sequenced using on an Illumina platform as previously described^18^. Briefly, genomic DNA was isolated with phenol–chloroform extraction. Fecal pellets were placed in 2 mL tubes containing 200 μL silica-zirconia beads (0.1 mm). Tubes were filled with 750 μL extraction buffer, 200 μL 20% SDS and 750 μL phenol–chloroform-isoamyl alcohol (25:24:1). After disruption, the aqueous phase was separated by centrifugation and washed twice with chloroform-isoamyl alcohol (24:1). The DNA was precipitated and resuspended in 10 mM Tris solution. The V3–V4 region of the 16S rRNA gene was amplified for 25 cycles using specific primers with adapter overhangs as per the Illumina library preparation guide. Forward primer: TCGTCGGCAGCGTCAGATGTGTATAAGAGACAGCCTACGGGNGGCWGCAG, Reverse primer: GTCTCGTGGGCTCGGAGATGTGTATAAGAGACAGGACTACHVGGGTATCTAATCC. Following purification of the PCR products, individual indexes were added to the amplicons by PCR. The amplicons were purified, pooled in equal quantities, and then sequenced on the Illumina MiSeq platform. The sequencing data have been deposited in the National Center for Biotechnology Information (NCBI) Gene Expression Omnibus (GEO) and is accessible through GEO Series accession number GSE153118.

### Read QC, trimming, filtering, denoising, and taxonomic assignment

Reads from demultiplexed FASTQ files were processed with the DADA2 pipeline^47^. Forward (R1) and reverse (R2) reads were truncated at 300 bp and 250 bp. The maximum number of expected errors for each read was set to 10 and the “trunQ” argument was set to 2 for read filtering. DADA2 was also used to perform error rate learning, dereplication, sample inference, read pair merging, and chimeric sequence removal with the “consensus” method. This resulted in 2,295 unique amplicon sequence variants (ASVs). Taxonomy was determined with a manually curated table based on the Silva^48^, GTDB^49^, Refseq^50^, and Greengenes^51^ databases. DADA2 was first used to match ASVs with formatted taxonomy references provided by Dada2 developers (Dec. 12, 2019) to infer taxonomic annotation. Taxonomic annotations from all four databases were directly compared to determine the most plausible taxonomy for each ASV. This achieved near-complete taxonomic annotation of bacterial phylum, class, order, family, genus, and species. Any ASVs with ambiguous or absent taxonomic information were given a designation that includes the next available taxonomic identifier. For example, if an ASV belonging to the Lactobacillaceae family is ambiguous at the genus rank, then it will be designated, “Lactobacillaceae_NA”. The final taxonomy table is available in supplementary table S4.

### Differential abundance testing and the ternary pattern mining

ASV count values were converted into a DESeq2 object for differential abundance testing using the R package “Phyloseq”^52^. The DESeq2 model: ~ Treatment + Time was used for pairwise differential abundance testing. Two main pairwise testing frameworks were applied to test for differential abundance. The first involved cross-treatment comparisons for each time point (− 2, 8, 14, 19, 29). Naïve mice were compared to CFA and EAE mice at corresponding time points. CFA and EAE mice were also directly compared at the same time points. The next testing framework involved pairwise differential abundance testing of each subsequent time point after immunization (8, 14, 19, 29) to the pre-immunization time point. This was performed within the groups for naïve, CFA, and EAE mice.

Differential abundance test results were converted into ternary sequences because it is a convenient format for quick interpretation and other downstream tests. In order to identify the broadest set of ASVs and taxa possible, a result from differential abundance testing was considered significant if the adjusted p-value was below an alpha threshold of 0.05. Differential abundance test results were translated into 0's if the DESeq2 adjusted p-value was greater than or equal to 0.05 and translated into 1 or − 1 if the adjusted p-value was less than 0.05 and the estimated log2-transformed fold change value was above or below 0. The resulting sequences allow for differential abundance test results to be quickly sorted into categories and directly compared. This was done to identify taxa that follow particular patterns over time or taxa that differ in relative abundance among treatment groups at specific time points. Differential abundance testing was performed on ASVs and on taxa at each taxonomic rank. The Phyloseq R package was used to agglomerate ASV abundances by taxonomy before differential abundance testing.

### Permutation testing and network construction

A permutation-renormalization-bootstrap network construction strategy called ReBoot^20^ was used to create bacterial co-abundance networks to study how EAE affects microbial co-occurrence and co-exclusionary relationships. Non-normalized ASV abundances were supplied to CoNet^21^, a Java Cytoscape plug-in and implementation of the ReBoot algorithm. Three networks were independently constructed by splitting the ASV abundance matrix by treatment group; naïve, CFA, and MOG immunized mice. The split abundance matrices were then imported into the Cytoscape. “Row_minocc” was set to 3 so that all ASVs appearing in less than 3 samples were removed. “Standardization” was set to “col_Norm” so abundances were normalized by sample read count.

CoNet allows for creation of an ensemble network and five default metrics for correlation strength were used. They include the Spearman and Pearson correlation coefficients, the Mutual information Score, and the Bray–Curtis and Kullback–Leibler Dissimilarity measures. The “Automatic Thresholding” was set to “edgeNumber” to retain the top and bottom 1,500 edges. In CoNet’s randomization menu, the “Routine” option was set to “edgeScores”, “Resampling” was set to “shuffle_Rows”, “Renormalize” was turned on, and P-value merge was set to “none”. These settings were used to run the bootstrap stage of the experiment over 100 random iterations to create the bootstrap distribution.

The bootstrap distributions were provided to CoNet the permutation stage of the experiment. These bootstrap distributions were compared against corresponding null distributions created by permuting the original data with a paired-variance Z-test. The resulting p-values were corrected using the Benjamini–Hochberg FDR procedure. If the p-value for a particular ASV⇔ASV pair was above 0.05, then the co-abundance/co-exclusionary relationship between the two ASVs was considered non-significant. P-values were calculated for each correlation metric. If at least two of the five metrics suggested significant co-abundance or mutual exclusion between two ASVs then that relationship was kept in the final network to be represented as an edge. The iGraph implementation of the Louvain algorithm was applied to identify communities within each network so that the modularity score of each ASV was maximized within a given network. Community significance was confirmed with a Wilcoxon Rank-Sum test.

### Taxonomic over representation analysis

Over-representation analysis with Fisher’s exact test was performed on the co-occurrence networks at each taxonomic rank (phylum, class, order, family, genus, species). This test was applied to the communities in each network detected by the Louvain algorithm. At each taxonomic rank, over-representation of a taxon within a community was calculated with a 2 × 2 contingency table that divided nodes into within versus outside a community and belonging to the taxon versus not belonging. A p-value was calculated for each taxon-community pair in a given network. This procedure was applied at all taxonomic ranks. Benjamini–Hochberg p-value correction was applied using all p-values discovered at a given taxonomic rank, setting n equal to the number of taxa in a given taxonomic rank multiplied by the number of eligible communities within a given network.

Testing for the over-representation of unique taxon-taxon relationships was accomplished by creating a subgraph from the network that consisted exclusively of the nodes from a given community. A network that excluded this subgraph was also created. A 2 × 2 contingency table was constructed by dividing all edges between membership status to a specific taxon-taxon relationship and membership status to the newly created community subgraph. A Benjamini–Hochberg p-value correction was applied in base R using all p-values discovered at a given taxonomic rank and setting n equal to the number of edge types (any unique pairing of two taxa) multiplied by the number of eligible communities within each network.

Fisher’s exact test was also used to test for associations between bacterial taxa and the ternary patterns created by differential abundance testing. This was accomplished by testing for enrichment of ASVs in certain ternary patterns within a given taxon of interest. A 2 × 2 contingency table was constructed by dividing ASVs by taxonomic membership and ternary pattern membership. This test was applied at all taxonomic ranks and over every observed ternary pattern. Benjamini–Hochberg p-value correction was applied using all p-values discovered at a given taxonomic rank, setting n equal to the number of taxa being tested multiplied by the number of observed ternary patterns. All applications of Fisher’s exact test were applied with a one-sided alternative hypothesis.

### PICRUSt2 metagenome imputation analysis

Samples were processed with PICRUSt2 to impute the putative metagenomic functional compositions of samples based on ASV abundances^23^. PICRUSt2 is a program that uses ASV abundances to impute enzyme and metabolic process abundances in a given sample. Enzyme Commission (EC) numbers and the pathways that they belong to can be estimated with this method. Raw ASV count data was imported into the Python programming environment and run through the PICRUSt2 pipeline with default parameters. The output is a matrix of samples as column and individual EC terms (or pathways) as rows. This output was used for downstream analyses including WGCNA (weighted gene correlation network analysis), principal component analysis (PCA) and differential abundance testing with DESeq2.

### EC and pathway WGCNA

WGCNA^10^ was performed on PICRUSt2-imputed MetaCyc Enzyme Consortium pathway and accession abundance datasets according to prescribed default settings. Abundance tables were normalized with DESeq2 using the model ~ Treatment + Time prior to WGCNA. These datasets were used to create signed network adjacency matrices with a soft thresholding power of 17 for EC pathways and 27 for EC accessions. The adjacency matrix was used to create a Topology Overlap Matrix, which was used to perform Hierarchical clustering with the UPGMA algorithm. ASV Clusters were sorted into network modules after hybrid adaptive branch pruning with the cutreeDynamic wrapper function. The “deepSplit” argument was set to 2, “pamRespectsDendro” was set to FALSE, and the default minimum cluster size was left at 30 for both networks. Module pairs were merged if they had an “eigengene” cross-correlation greater than 0.7 (a minimum dissimilarity of 0.3). Eigengenes are defined as being the first/primary principle component of the nodes in a given network module across all samples used in network construction. The resulting networks were evaluated for correlation strengths between module eigengenes and sample phenotype data.

## Supplementary information

Supplementary Information 1.

Supplementary Information 2.
