# Supplementary material for: Experimental autoimmune encephalomyelitis is associated with changes of the microbiota composition in the gastrointestinal tract
Source: Sci Rep. 2020 Sep 16;10:15183. doi: 10.1038/s41598-020-72197-y (PMC7494894; doi:10.1038/s41598-020-72197-y)
Supplement: Supplementary file 2 — Supplementary Information 2. [file 41598_2020_72197_MOESM2_ESM.pdf]

Supplementary figures for:

Experimental autoimmune encephalomyelitis is associated with changes of the microbiota composition in the gastrointestinal tract.

David M. Johanson II[1,2], Jennifer E. Goertz[1,2,3], Ioana A. Marin[1,2,4], John Costello[1,2], Christopher C. Overall[1,2,5] and Alban Gaultier[1,2,*]

[1]Center for Brain Immunology and Glia, [2]Department of Neuroscience, School of Medicine, University of Virginia, Charlottesville, VA 22908, [3]Cornell University, Dept. of Neuroscience, Ithaca, NY, 14850, [4]Stanford University, Dept. of Neuroscience, Stanford, CA, 94305, [5]Myriad Genetics, Inc., San Francisco, CA, 94080.

S1

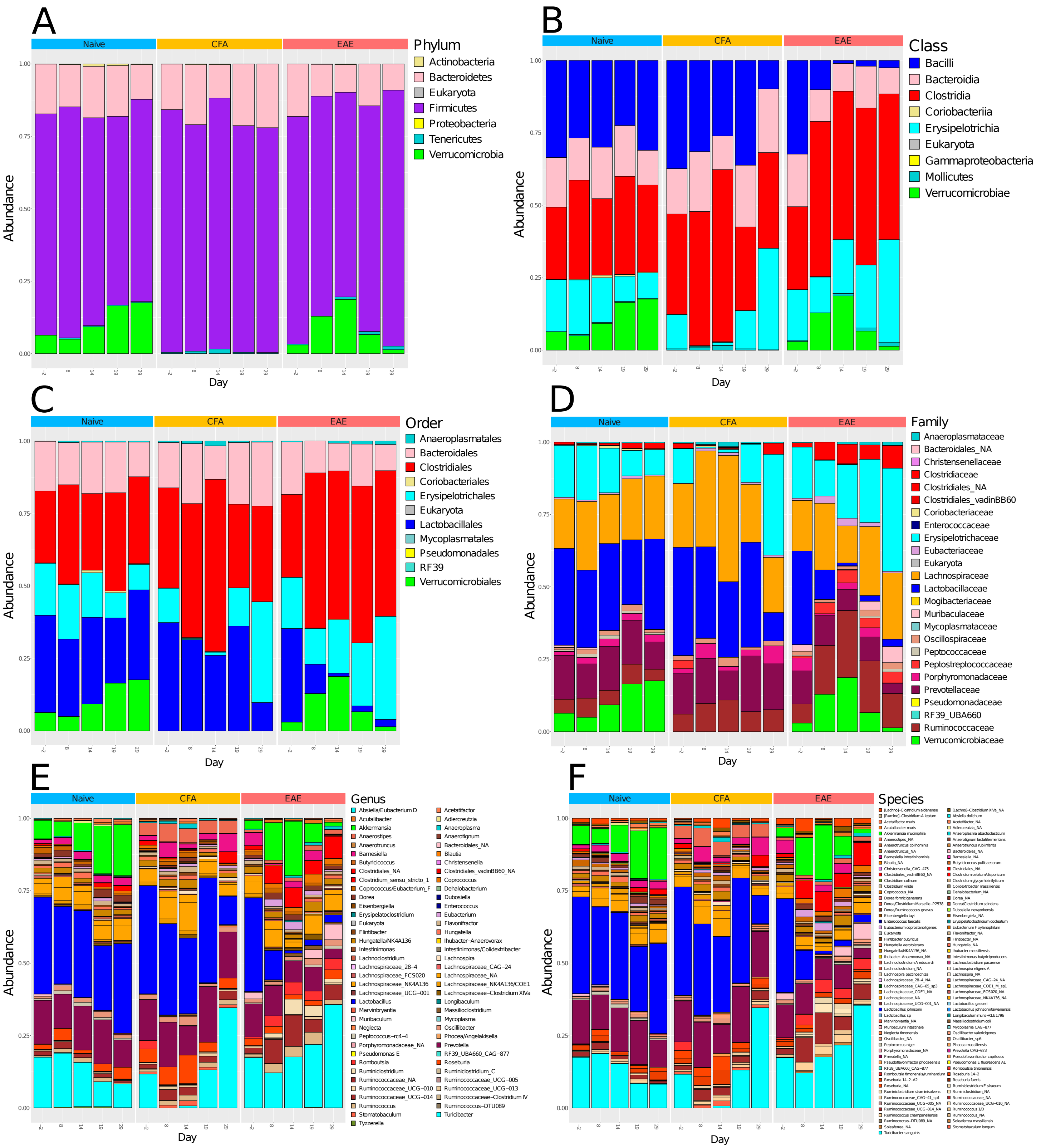

A
Naive
CFA
EAE
Abundance
Day
Phylum
Actinobacteria
Bacteroidetes
Eukaryota
Firmicutes
Proteobacteria
Tenericutes
Verrucomicrobia
B
Class
Bacilli
Bacteroidia
Clostridia
Coriobacteriia
Erysipelotrichia
Eukaryota
Gammaproteobacteria
Mollicutes
Verrucomicrobiae
C
Order
Anaeroplasmatales
Bacteroidales
Clostridiales
Coriobacteriales
Erysipelotrichales
Eukaryota
Lactobacillales
Mycoplasmatales
Pseudomonadales
RF39
Verrucomicrobiales
D
Family
Anaeroplasmataceae
Bacteroidales_NA
Christensenellaceae
Clostridiaceae
Clostridiales_NA
Clostridiales_vadinBB60
Coriobacteriaceae
Enterococcaceae
Erysipelotrichaceae
Eubacteriaceae
Eukaryota
Lachnospiraceae
Lactobacillaceae
Mogibacteriaceae
Muribaculaceae
Mycoplasmataceae
Oscillospiraceae
Peptococcaceae
Peptostreptococcaceae
Porphyromonadaceae
Prevotellaceae
Pseudomonadaceae
RF39_UBA660
Ruminococcaceae
Verrucomicrobiaceae
E
Genus
F
Species

Figure S1: Stacked barplots of the relative abundances of every identified bacterial taxon at each taxonomic rank level. Relative abundances were calculated from raw ASV abundances that had been grouped at each taxonomic level. For each taxonomic rank level, taxa are stacked in alphabetical order as they appear in their respective legends. Each facet represents a different treatment group (naïve, CFA, EAE) and each bar includes all samples that belong to the time point represented on the x-axis. **A**: Phylum rank relative abundances. **B**: Class rank relative abundances. **C**: Order rank relative abundances. **D**: Family rank relative abundances. **E**: Genus rank relative abundances. **F**: Species rank relative abundances.

S2

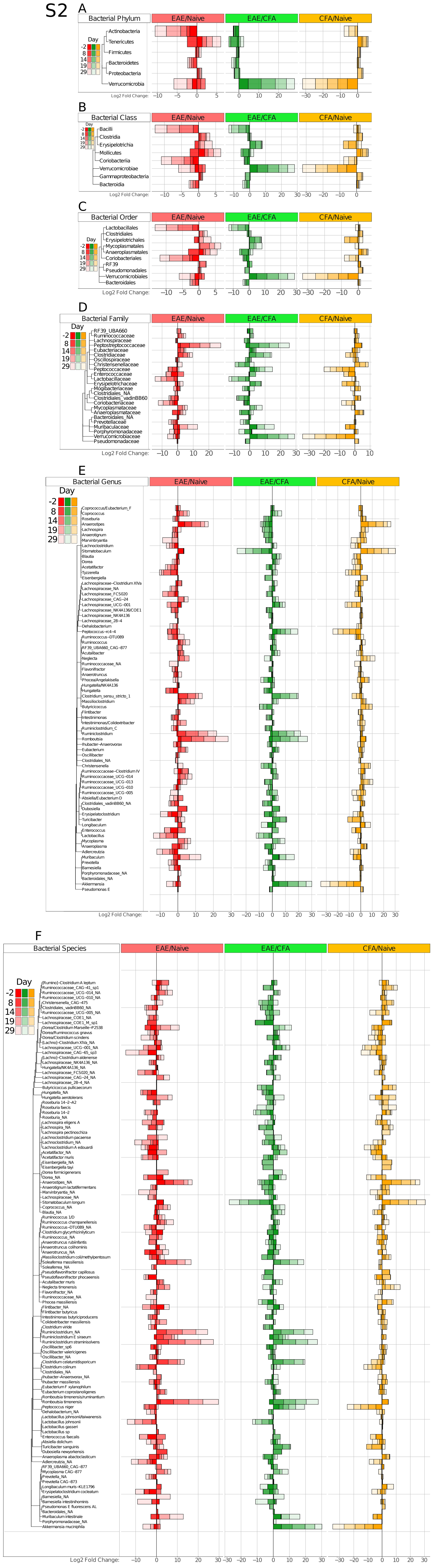
A
Bacterial Phylum
EAE/Naive
EAE/CFA
CFA/Naive
Day
-2
8
14
19
29
Actinobacteria
Tenericutes
Firmicutes
Bacteroidetes
Proteobacteria
Verrucomicrobia
Log2 Fold Change:
B
Bacterial Class
Bacilli
Clostridia
Erysipelotrichia
Mollicutes
Coriobacteriia
Verrucomicrobiae
Gammaproteobacteria
Bacteroidia
C
Bacterial Order
Lactobacillales
Clostridiales
Erysipelotrichales
Mycoplasmatales
Anaeroplasmatales
Coriobacteriales
RF39
Pseudomonadales
Verrucomicrobiales
Bacteroidales
D
Bacterial Family
RF39_UBA660
Ruminococcaceae
Lachnospiraceae
Peptostreptococcaceae
Eubacteriaceae
Clostridiaceae
Oscillospiraceae
Christensenellaceae
Peptococcaceae
Enterococcaceae
Lactobacillaceae
Erysipelotrichaceae
Mogibacteriaceae
Clostridiales_NA
Clostridiales_vadinBB60
Coriobacteriaceae
Mycoplasmataceae
Anaeroplasmataceae
Bacteroidales_NA
Prevotellaceae
Muribaculaceae
Porphyromonadaceae
Verrucomicrobiaceae
Pseudomonadaceae
E
Bacterial Genus
Coprococcus/Eubacterium_F
Coprococcus
Roseburia
Anaerostipes
Lachnospira
Anaerotignum
Marvinbryantia
Lachnoclostridium
Stomatobaculum
Blautia
Dorea
Acetatifactor
Tyzzerella
Eisenbergiella
Lachnospiraceae–Clostridium XIVa
Lachnospiraceae_NA
Lachnospiraceae_FCS020
Lachnospiraceae_CAG–24
Lachnospiraceae_UCG–001
Lachnospiraceae_NK4A136/COE1
Lachnospiraceae_NK4A136
Lachnospiraceae_28–4
Dehalobacterium
Peptococcus–rc4–4
Ruminococcus–DTU089
Ruminococcus
RF39_UBA660_CAG–877
Acutalibacter
Neglecta
Ruminococcaceae_NA
Flavonifractor
Anaerotruncus
Phocea/Angelakisella
Hungatella/NK4A136
Hungatella
Clostridium_sensu_stricto_1
Massilioclostridium
Butyricicoccus
Flintibacter
Intestinimonas
Intestinimonas/Colidextribacter
Ruminiclostridium_C
Ruminiclostridium
Romboutsia
Ihubacter–Anaerovorax
Eubacterium
Oscillibacter
Clostridiales_NA
Christensenella
Ruminococcaceae–Clostridium IV
Ruminococcaceae_UCG–014
Ruminococcaceae_UCG–013
Ruminococcaceae_UCG–010
Ruminococcaceae_UCG–005
Absiella/Eubacterium D
Clostridiales_vadinBB60_NA
Dubosiella
Erysipelatoclostridium
Turicibacter
Longibaculum
Enterococcus
Lactobacillus
Mycoplasma
Anaeroplasma
Adlercreutzia
Muribaculum
Prevotella
Barnesiella
Porphyromonadaceae_NA
Bacteroidales_NA
Akkermansia
Pseudomonas E
F
Bacterial Species
(Rumino)–Clostridium A leptum
Ruminococcaceae_CAG–41_sp1
Ruminococcaceae_UCG–014_NA
Ruminococcaceae_UCG–010_NA
Christensenella_CAG–475
Clostridiales_vadinBB60_NA
Ruminococcaceae_UCG–005_NA
Lachnospiraceae_COE1_NA
Lachnospiraceae_COE1_M_sp1
Dorea/Clostridium Marseille–P2538
Dorea/Ruminococcus gnavus
Dorea/Clostridium scindens
(Lachno)–Clostridium XIVa_NA
Lachnospiraceae_UCG–001_NA
Lachnospiraceae_CAG–65_sp3
(Lachno)–Clostridium aldenense
Lachnospiraceae_NK4A136_NA
Hungatella/NK4A136_NA
Lachnospiraceae_FCS020_NA
Lachnospiraceae_CAG–24_NA
Lachnospiraceae_28–4_NA
Butyricicoccus pullicaecorum
Hungatella_NA
Hungatella aerotolerans
Roseburia 14–2–A2
Roseburia faecis
Roseburia 14–2
Roseburia_NA
Lachnospira eligens A
Lachnospira_NA
Lachnospira pectinoschiza
Lachnoclostridium pacaense
Lachnoclostridium_NA
Lachnoclostridium A edouardi
Acetatifactor_NA
Acetatifactor muris
Eisenbergiella_NA
Eisenbergiella tayi
Dorea formicigenerans
Dorea_NA
Anaerostipes_NA
Anaerotignum lactatifermentans
Marvinbryantia_NA
Lachnospiraceae_NA
Stomatobaculum longum
Coprococcus_NA
Blautia_NA
Ruminococcus 1/D
Ruminococcus champanellensis
Ruminococcus–DTU089_NA
Clostridium glycyrrhizinilyticum
Ruminococcus_NA
Anaerotruncus rubiinfantis
Anaerotruncus colihominis
Anaerotruncus_NA
Massilioclostridium coli/methylpentosum
Solearferrea massiliensis
Soleaferrea_NA
Pseudoflavonifractor capillosus
Pseudoflavonifractor phocaeensis
Acutalibacter muris
Neglecta timonensis
Flavonifractor_NA
Ruminococcaceae_NA
Phocea massiliensis
Flintibacter_NA
Flintibacter butyricus
Intestinimonas butyriciproducens
Colidextribacter massiliensis
Clostridium viride
Ruminiclostridium_NA
Ruminiclostridium E siraeum
Ruminiclostridium straminisolvens
Oscillibacter_sp6
Oscillibacter valericigenes
Oscillibacter_NA
Clostridium celatum/disporicum
Clostridium colinum
Clostridiales_NA
Ihubacter–Anaerovorax_NA
Ihubacter massiliensis
Eubacterium F xylanophilum
Eubacterium coprostanoligenes
Romboutsia timonensis/ruminantium
Romboutsia timonensis
Peptococcus niger
Dehalobacterium_NA
Lactobacillus johnsonii/taiwanensis
Lactobacillus johnsonii
Lactobacillus gasseri
Lactobacillus sp
Enterococcus faecalis
Absiella dolichum
Turicibacter sanguinis
Dubosiella newyorkensis
Anaeroplasma abactoclasticum
Adlercreutzia_NA
RF39_UBA660_CAG–877
Mycoplasma CAG–877
Prevotella_NA
Prevotella CAG–873
Longibaculum muris–KLE1796
Erysipelatoclostridium cocleatum
Barnesiella_NA
Barnesiella intestinihominis
Pseudomonas E fluorescens AL
Bacteroidales_NA
Muribaculum intestinale
Porphyromonadaceae_NA
Akkermansia muciniphila
Log2 Fold Change:

Figure S2: Stacked barplots of DESeq2 log2 fold change values for cross-treatment pairwise comparisons that have been applied independently at each taxonomic rank level. Each bar represents an individual pairwise comparison between two experimental groups (EAE/naïve, EAE/CFA, CFA/naïve) that was made at a specific time point (-2, 8, 14, 19, and 29 dpi). Trees represent the approximate taxonomy and were determined by manual annotation and supplementation of NCBI taxonomy. **A**: Phylum rank stacked barplot. **B**: Class rank stacked barplot. **C**: Order rank stacked barplot. **D**: Family rank stacked barplot. **E**: Genus rank stacked barplot. **F**: Species rank stacked barplot.

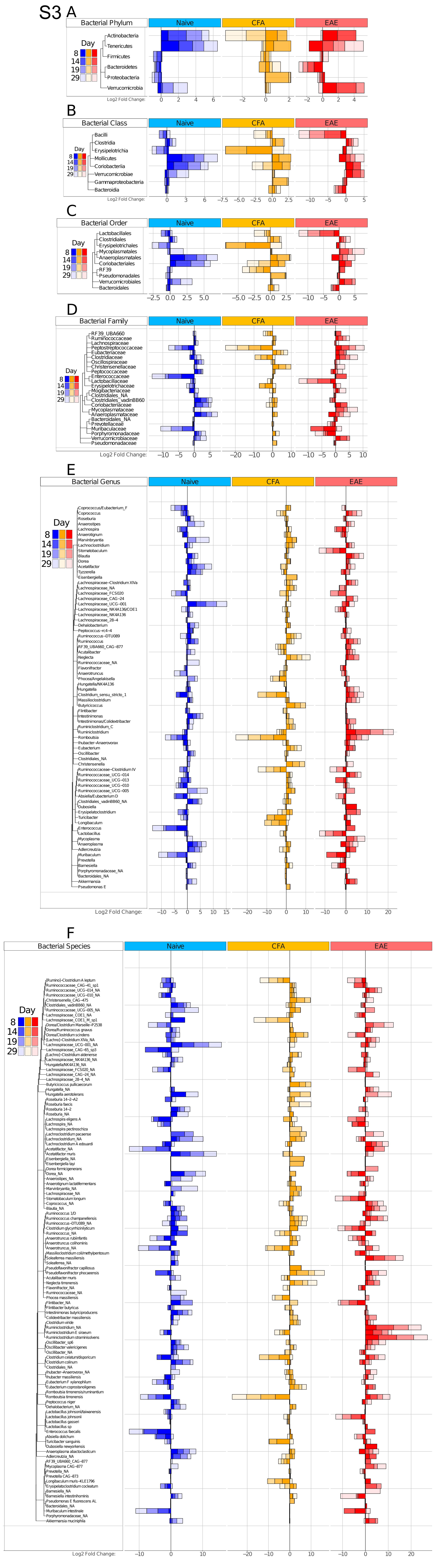
S3 A
Bacterial Phylum
Naive
CFA
EAE
Day
8
14
19
29
Actinobacteria
Tenericutes
Firmicutes
Bacteroidetes
Proteobacteria
Verrucomicrobia
Log2 Fold Change:
B
Bacterial Class
Bacilli
Clostridia
Erysipelotrichia
Mollicutes
Coriobacteriia
Verrucomicrobiae
Gammaproteobacteria
Bacteroidia
C
Bacterial Order
Lactobacillales
Clostridiales
Erysipelotrichales
Mycoplasmatales
Anaeroplasmatales
Coriobacteriales
RF39
Pseudomonadales
Verrucomicrobiales
Bacteroidales
D
Bacterial Family
E
Bacterial Genus
F
Bacterial Species

Figure S3: Stacked barplots of DESeq2 log2 fold change values for pairwise comparisons within experimental groups but across time that have been applied independently at each taxonomic rank level. Each bar represents an individual pairwise comparison performed within an experimental group (naïve, CFA, or EAE) between the pre-immunization time point (-2 dpi) and a subsequent time point (8, 14, 19, 29 dpi). Trees represent the approximate taxonomy and were determined by manual annotation and supplementation of NCBI taxonomy. **A**: Phylum rank stacked barplot. **B**: Class rank stacked barplot. **C**: Order rank stacked barplot. **D**: Family rank stacked barplot. **E**: Genus rank stacked barplot. **F**: Species rank stacked barplot.

S4

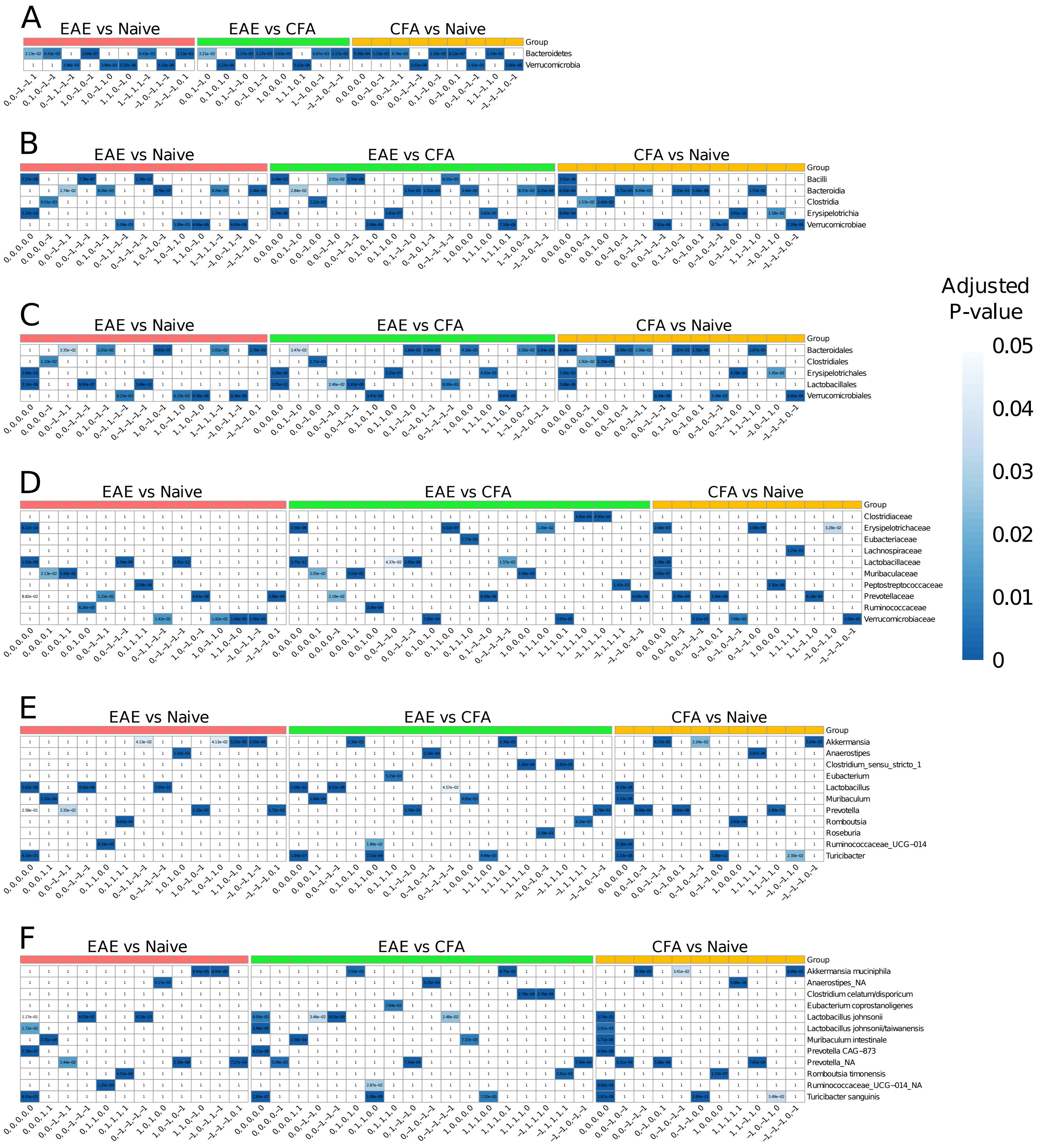
A
EAE vs Naive
EAE vs CFA
CFA vs Naive
Group
Bacteroidetes
Verrucomicrobia
B
Bacilli
Bacteroidia
Clostridia
Erysipelotrichia
Verrucomicrobiae
C
Bacteroidales
Clostridiales
Erysipelotrichales
Lactobacillales
Verrucomicrobiales
D
Clostridiaceae
Erysipelotrichaceae
Eubacteriaceae
Lachnospiraceae
Lactobacillaceae
Muribaculaceae
Peptostreptococcaceae
Prevotellaceae
Ruminococcaceae
Verrucomicrobiaceae
E
Akkermansia
Anaerostipes
Clostridium_sensu_stricto_1
Eubacterium
Lactobacillus
Muribaculum
Prevotella
Romboutsia
Roseburia
Ruminococcaceae_UCG−014
Turicibacter
F
Akkermansia muciniphila
Anaerostipes_NA
Clostridium celatum/disporicum
Eubacterium coprostanoligenes
Lactobacillus johnsonii
Lactobacillus johnsonii/taiwanensis
Muribaculum intestinale
Prevotella CAG−873
Prevotella_NA
Romboutsia timonensis
Ruminococcaceae_UCG−014_NA
Turicibacter sanguinis
Adjusted P-value
0.05
0.04
0.03
0.02
0.01
0

Figure S4: Heatmaps of taxonomic over-representation analysis results at each taxonomic rank level. Cell color is scaled from 0 to 0.05 and represents the p-value derived from Fisher's exact test for enrichment of a ternary pattern within a taxon. Ternary patterns represented include all cross-treatment pairwise comparisons (EAE/naïve, EAE/CFA, and CFA/naïve at -2, 8, 14, 19, and 29 dpi). P-values have been corrected with the BH procedure. Columns represent unique ternary patterns and rows represent unique taxa. Heatmaps are subsets of full results that include only taxa and patterns that possess at least one significant P-value. **A**: Phylum rank taxonomic over-representation heatmap. **B**: Class rank taxonomic over-representation heatmap. **C**: Order rank taxonomic over-representation heatmap. **D**: Family rank taxonomic over-representation heatmap. **E**: Genus rank taxonomic over-representation heatmap. **F**: Species rank taxonomic over-representation heatmap.

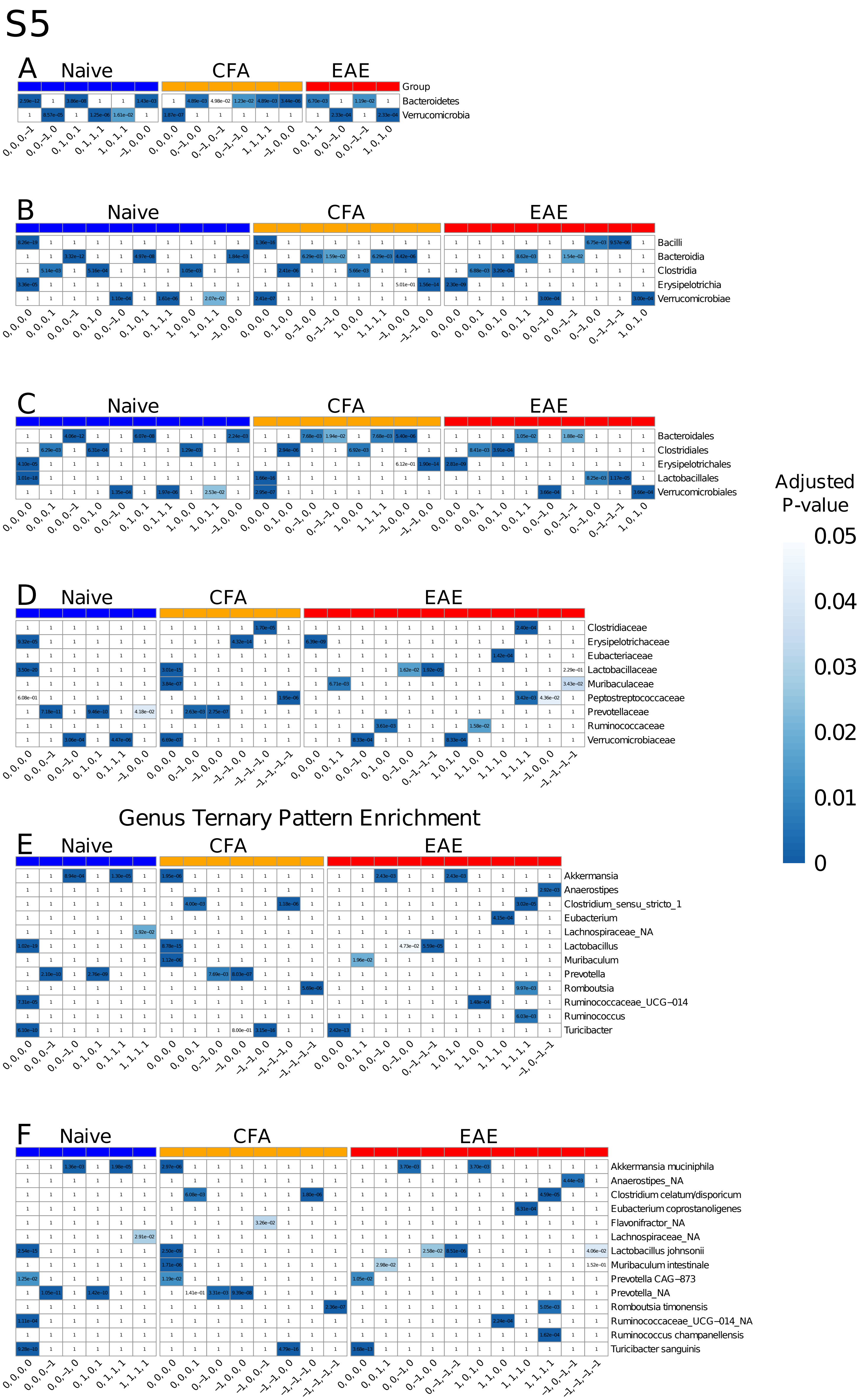
S5
A
Naive
CFA
EAE
Group
Bacteroidetes
Verrucomicrobia
B
Bacilli
Bacteroidia
Clostridia
Erysipelotrichia
Verrucomicrobiae
C
Bacteroidales
Clostridiales
Erysipelotrichales
Lactobacillales
Verrucomicrobiales
D
Clostridiaceae
Erysipelotrichaceae
Eubacteriaceae
Lactobacillaceae
Muribaculaceae
Peptostreptococcaceae
Prevotellaceae
Ruminococcaceae
Verrucomicrobiaceae
Genus Ternary Pattern Enrichment
E
Akkermansia
Anaerostipes
Clostridium_sensu_stricto_1
Eubacterium
Lachnospiraceae_NA
Lactobacillus
Muribaculum
Prevotella
Romboutsia
Ruminococcaceae_UCG−014
Ruminococcus
Turicibacter
F
Akkermansia muciniphila
Anaerostipes_NA
Clostridium celatum/disporicum
Eubacterium coprostanoligenes
Flavonifractor_NA
Lachnospiraceae_NA
Lactobacillus johnsonii
Muribaculum intestinale
Prevotella CAG−873
Prevotella_NA
Romboutsia timonensis
Ruminococcaceae_UCG−014_NA
Ruminococcus champanellensis
Turicibacter sanguinis
Adjusted
P-value
0.05
0.04
0.03
0.02
0.01
0

Figure S5: Heatmaps of taxonomic over-representation analysis results at each taxonomic rank level. Cell color is scaled from 0 to 0.05 and represents the p-value derived from Fisher's exact test for enrichment of a ternary pattern within a taxon. Ternary patterns represented include all within-treatment pairwise comparisons (8/-2, 14/-2, 19/-2, and 29/-2 for naïve, CFA, and EAE). P-values have been corrected with the BH procedure. Columns represent unique ternary patterns and rows represent unique taxa. Heatmaps are subsets of full results that include only taxa and patterns that possess at least one significant P-value. **A**: Phylum rank taxonomic over-representation heatmap. **B**: Class rank taxonomic over-representation heatmap. **C**: Order rank taxonomic over-representation heatmap. **D**: Family rank taxonomic over-representation heatmap. **E**: Genus rank taxonomic over-representation heatmap. **F**: Species rank taxonomic over-representation heatmap.

S6

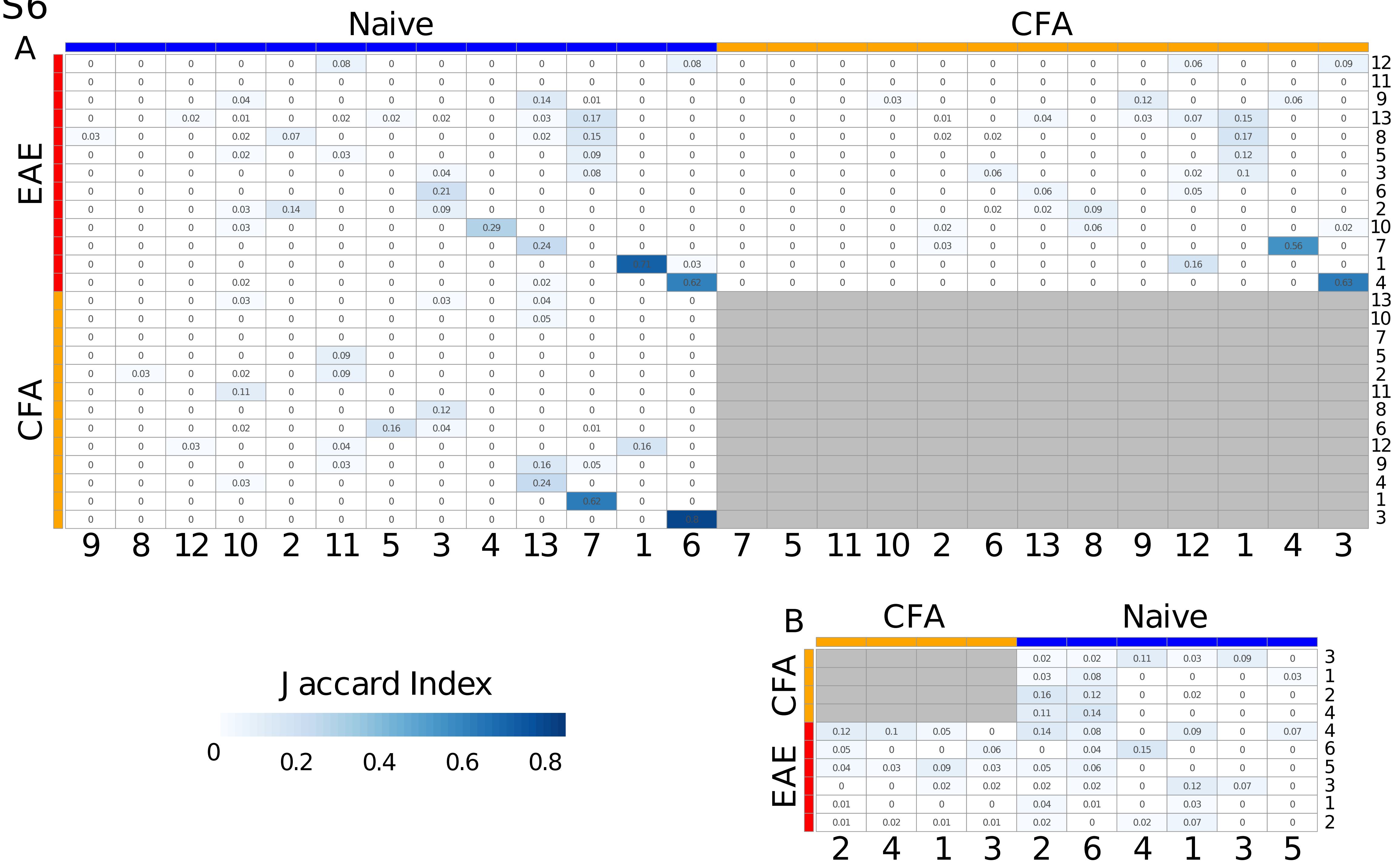
A
Naive
CFA
EAE
CFA
9 8 12 10 2 11 5 3 4 13 7 1 6 7 5 11 10 2 6 13 8 9 12 1 4 3
12 11 9 13 8 5 3 6 2 10 7 1 4 13 10 7 5 2 11 8 6 12 9 4 1 3
B
CFA
Naive
CFA
EAE
2 4 1 3 2 6 4 1 3 5
3 1 2 4 4 6 5 3 1 2
Jaccard Index
0
0.2
0.4
0.6
0.8

Figure S6: Heatmaps of Jaccard Indices for community node composition among the co-occurrence and mutual exclusion networks of each treatment group (naïve, CFA, EAE). Jaccard Index was calculated using the unique identities of the ASVs that make up the communities in each network. **A**: Jaccard Index heatmap for co-occurrence network pairwise comparisons. Cell color and number is the Jaccard Index, denoting degree of node identity overlap for the two communities being compared. **B**: Jaccard Index heatmap for mutual exclusion network pairwise comparisons. Follows same convention as S6A.

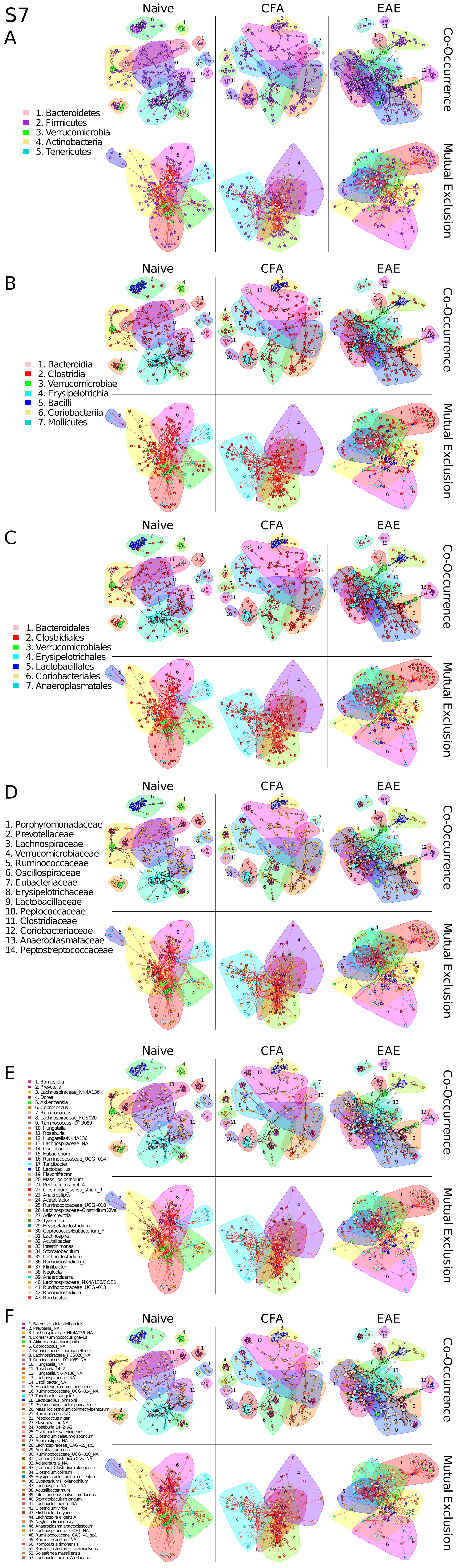

S7
A
Naive
CFA
EAE
Co-Occurrence
Mutual Exclusion
1. Bacteroidetes
2. Firmicutes
3. Verrucomicrobia
4. Actinobacteria
5. Tenericutes
B
1. Bacteroidia
2. Clostridia
3. Verrucomicrobiae
4. Erysipelotrichia
5. Bacilli
6. Coriobacteriia
7. Mollicutes
C
1. Bacteroidales
2. Clostridiales
3. Verrucomicrobiales
4. Erysipelotrichales
5. Lactobacillales
6. Coriobacteriales
7. Anaeroplasmatales
D
1. Porphyromonadaceae
2. Prevotellaceae
3. Lachnospiraceae
4. Verrucomicrobiaceae
5. Ruminococcaceae
6. Oscillospiraceae
7. Eubacteriaceae
8. Erysipelotrichaceae
9. Lactobacillaceae
10. Peptococcaceae
11. Clostridiaceae
12. Coriobacteriaceae
13. Anaeroplasmataceae
14. Peptostreptococcaceae
E
1. Barnesiella
2. Prevotella
3. Lachnospiraceae_NK4A136
4. Dorea
5. Akkermansia
6. Coprococcus
7. Ruminococcus
8. Lachnospiraceae_FCS020
9. Ruminococcus–DTU089
10. Hungatella
11. Roseburia
12. Hungatella/NK4A136
13. Lachnospiraceae_NA
14. Oscillibacter
15. Eubacterium
16. Ruminococcaceae_UCG–014
17. Turicibacter
18. Lactobacillus
19. Flavonifractor
20. Massilioclostridium
21. Peptococcus–rc4–4
22. Clostridium_sensu_stricto_1
23. Anaerostipes
24. Acetatifactor
25. Ruminococcaceae_UCG–010
26. Lachnospiraceae–Clostridium XIVa
27. Adlercreutzia
28. Tyzzerella
29. Erysipelatoclostridium
30. Coprococcus/Eubacterium_F
31. Lachnospira
32. Acutalibacter
33. Intestinimonas
34. Stomatobaculum
35. Lachnoclostridium
36. Ruminiclostridium_C
37. Flintibacter
38. Neglecta
39. Anaeroplasma
40. Lachnospiraceae_NK4A136/COE1
41. Ruminococcaceae_UCG–013
42. Ruminiclostridium
43. Romboutsia
F
1. Barnesiella intestinihominis
2. Prevotella_NA
3. Lachnospiraceae_NK4A136_NA
4. Dorea/Ruminococcus gnavus
5. Akkermansia muciniphila
6. Coprococcus_NA
7. Ruminococcus champanellensis
8. Lachnospiraceae_FCS020_NA
9. Ruminococcus–DTU089_NA
10. Hungatella_NA
11. Roseburia 14–2
12. Hungatella/NK4A136_NA
13. Lachnospiraceae_NA
14. Oscillibacter_NA
15. Eubacterium coprostanoligenes
16. Ruminococcaceae_UCG–014_NA
17. Turicibacter sanguinis
18. Lactobacillus johnsonii
19. Pseudoflavonifractor phocaeensis
20. Massilioclostridium coli/methylpentosum
21. Ruminococcus 1/D
22. Peptococcus niger
23. Flavonifractor_NA
24. Roseburia 14–2–A2
25. Oscillibacter valericigenes
26. Clostridium celatum/disporicum
27. Anaerostipes_NA
28. Lachnospiraceae_CAG–65_sp3
29. Acetatifactor muris
30. Ruminococcaceae_UCG–010_NA
31. [Lachno]–Clostridium XlVa_NA
32. Adlercreutzia_NA
33. [Lachno]–Clostridium aldenense
34. Clostridium colinum
35. Erysipelatoclostridium cocleatum
36. Eubacterium F xylanophilum
37. Lachnospira_NA
38. Acutalibacter muris
39. Intestinimonas butyriciproducens
40. Stomatobaculum longum
41. Lachnoclostridium_NA
42. Clostridium viride
43. Flintibacter butyricus
44. Lachnospira eligens A
45. Neglecta timonensis
46. Anaeroplasma abactoclasticum
47. Lachnospiraceae_COE1_NA
48. Ruminococcaceae_CAG–41_sp1
49. Ruminiclostridium_NA
50. Romboutsia timonensis
51. Ruminiclostridium straminisolvens
52. Soleaferrea massiliensis
53. Lachnoclostridium A edouardi

Figure S7: Network diagrams of co-occurrence and mutual exclusion networks labeled at different taxonomic ranks. **A**: Phylum rank labeled network diagrams. **B**: Class rank labeled network diagrams. **C**: Order rank labeled network diagrams. **D**: Family rank labeled network diagrams. **E**: Genus rank labeled network diagrams. **F**: Species rank labeled network diagrams.

S8

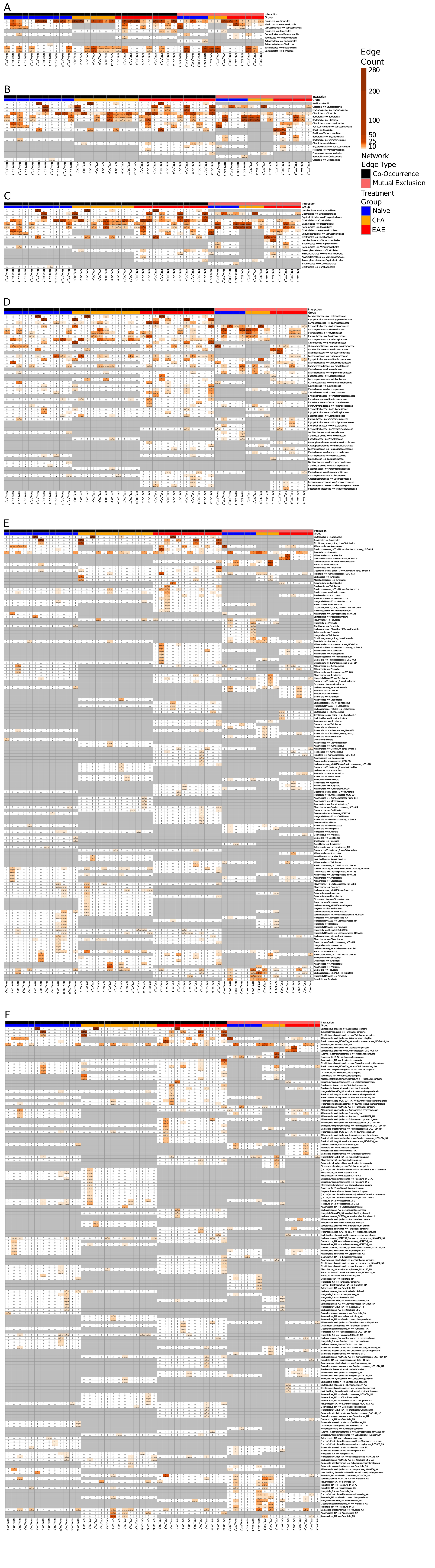
A
B
C
D
E
F
Edge Count
280
200
100
50
25
10
Network Edge Type
Co-Occurrence
Mutual Exclusion
Treatment Group
Naive
CFA
EAE

Figure S8: Heatmaps of BH-adjusted P-values for over-representation of unique edges within the communities of each co-occurrence network. Each row represents an edge; distinguished by the two distinct taxa it connects. Each column is a community in one of the three networks. Cell color corresponds to edge frequency within each network and each cell contains an edge frequency number followed by a significance level in parentheses (if no parentheses then n.s.). Heatmaps have been filtered to only contain communities with at least one significant P-value among the edges being displayed. Edges were filtered to contain at least one significant P-value representing over-representation of that edge in the corresponding community. Grey cells represent edges that were entirely absent from the network they appear under. Horizontal color bars represent network treatment identity (naïve, CFA, or EAE) and network type (co-occurrence or mutual exclusion) **A**: Phylum rank edge over-representation heatmap. **B**: Class rank edge over-representation heatmap. **C**: Order rank edge over-representation heatmap. **D**: Family rank edge over-representation heatmap. **E**: Genus rank edge over-representation heatmap. **F**: Species rank edge over-representation heatmap.

S9

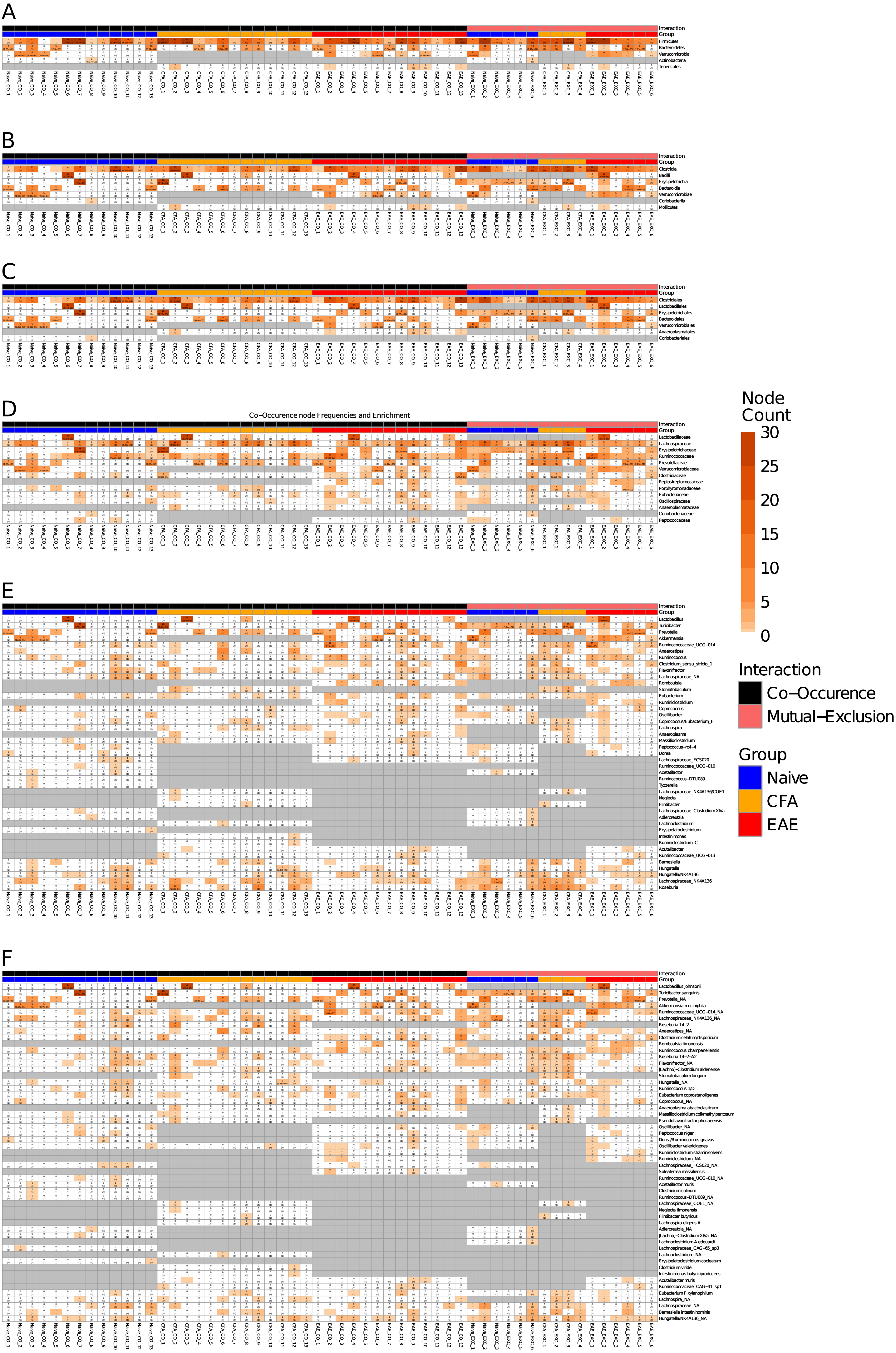
A
Interaction
Group
Firmicutes
Bacteroidetes
Verrucomicrobia
Actinobacteria
Tenericutes
B
Clostridia
Bacilli
Erysipelotrichia
Bacteroidia
Verrucomicrobiae
Coriobacteriia
Mollicutes
C
Clostridiales
Lactobacillales
Erysipelotrichales
Bacteroidales
Verrucomicrobiales
Anaeroplasmatales
Coriobacteriales
D
Co−Occurence node Frequencies and Enrichment
Lactobacillaceae
Lachnospiraceae
Erysipelotrichaceae
Ruminococcaceae
Prevotellaceae
Verrucomicrobiaceae
Clostridiaceae
Peptostreptococcaceae
Porphyromonadaceae
Eubacteriaceae
Oscillospiraceae
Anaeroplasmataceae
Coriobacteriaceae
Peptococcaceae
E
Lactobacillus
Turicibacter
Prevotella
Akkermansia
Ruminococcaceae_UCG−014
Anaerostipes
Ruminococcus
Clostridium_sensu_stricto_1
Flavonifractor
Lachnospiraceae_NA
Romboutsia
Stomatobaculum
Eubacterium
Ruminiclostridium
Coprococcus
Oscillibacter
Coprococcus/Eubacterium_F
Lachnospira
Anaeroplasma
Massiliclostridium
Peptococcus−rc4−4
Dorea
Lachnospiraceae_FCS020
Ruminococcaceae_UCG−010
Acetatifactor
Ruminococcus−DTU089
Tyzzerella
Lachnospiraceae_NK4A136/COE1
Neglecta
Flintibacter
Lachnospiraceae−Clostridium XIVa
Adlercreutzia
Lachnoclostridium
Erysipelatoclostridium
Intestinimonas
Ruminiclostridium_C
Acutalibacter
Ruminococcaceae_UCG−013
Barnesiella
Hungatella
Hungatella/NK4A136
Lachnospiraceae_NK4A136
Roseburia
F
Lactobacillus johnsonii
Turicibacter sanguinis
Prevotella_NA
Akkermansia muciniphila
Ruminococcaceae_UCG−014_NA
Lachnospiraceae_NK4A136_NA
Roseburia 14−2
Anaerostipes_NA
Clostridium celatum/disporicum
Romboutsia timonensis
Ruminococcus champanellensis
Roseburia 14−2−A2
Flavonifractor_NA
[Lachno]−Clostridium aldenense
Stomatobaculum longum
Hungatella_NA
Ruminococcus 1/D
Eubacterium coprostanoligenes
Coprococcus_NA
Anaeroplasma abactoclasticum
Massiliclostridium coli/methylpentosum
Pseudoflavonifractor phocaeensis
Oscillibacter_NA
Peptococcus niger
Dorea/Ruminococcus gnavus
Oscillibacter valericigenes
Ruminiclostridium straminisolvens
Ruminiclostridium_NA
Lachnospiraceae_FCS020_NA
Soleaferrea massiliensis
Ruminococcaceae_UCG−010_NA
Acetatifactor muris
Clostridium colinum
Ruminococcus−DTU089_NA
Lachnospiraceae_COE1_NA
Neglecta timonensis
Flintibacter butyricus
Lachnospira eligens A
Adlercreutzia_NA
[Lachno]−Clostridium XIVa_NA
Lachnoclostridium A edouardi
Lachnospiraceae_CAG−65_sp3
Lachnoclostridium_NA
Erysipelatoclostridium cocleatum
Clostridium viride
Intestinimonas butyriciproducens
Acutalibacter muris
Ruminococcaceae_CAG−41_sp1
Eubacterium F xylanophilum
Lachnospira_NA
Lachnospiraceae_NA
Barnesiella intestinihominis
Hungatella/NK4A136_NA
Naive_CO_1
Naive_CO_2
Naive_CO_3
Naive_CO_4
Naive_CO_5
Naive_CO_6
Naive_CO_7
Naive_CO_8
Naive_CO_9
Naive_CO_10
Naive_CO_11
Naive_CO_12
Naive_CO_13
CFA_CO_1
CFA_CO_2
CFA_CO_3
CFA_CO_4
CFA_CO_5
CFA_CO_6
CFA_CO_7
CFA_CO_8
CFA_CO_9
CFA_CO_10
CFA_CO_11
CFA_CO_12
CFA_CO_13
EAE_CO_1
EAE_CO_2
EAE_CO_3
EAE_CO_4
EAE_CO_5
EAE_CO_6
EAE_CO_7
EAE_CO_8
EAE_CO_9
EAE_CO_10
EAE_CO_11
EAE_CO_12
EAE_CO_13
Naive_EXC_1
Naive_EXC_2
Naive_EXC_3
Naive_EXC_4
Naive_EXC_5
Naive_EXC_6
CFA_EXC_1
CFA_EXC_2
CFA_EXC_3
CFA_EXC_4
EAE_EXC_1
EAE_EXC_2
EAE_EXC_3
EAE_EXC_4
EAE_EXC_5
EAE_EXC_6
Node Count
30
25
20
15
10
5
0
Interaction
Co−Occurence
Mutual−Exclusion
Group
Naive
CFA
EAE

Figure S9: Heatmaps of BH-adjusted P-values for over-representation of unique nodes within the communities of each co-occurrence network. Each row represents all nodes belonging to a given taxon. Each column is a community in one of the three networks. Cell color corresponds to edge frequency within each network and each cell contains a node frequency number followed by a significance level in parentheses (if no parentheses then n.s.). The heatmaps have been filtered to only contain communities with at least one significant P-value among the nodes being displayed. Nodes were filtered to contain at least one significant P-value representing over-representation of that node in the corresponding community. Grey cells represent taxa that were entirely absent from the network they appear under. Horizontal color bars represent network treatment identity (naïve, CFA, or EAE) and network type (co-occurrence or mutual exclusion). **A**: Phylum rank node over-representation heatmap. **B**: Class rank node over-representation heatmap. **C**: Order rank node over-representation heatmap. **D**: Family rank node over-representation heatmap. **E**: Genus rank node over-representation heatmap. **F**: Species rank node over-representation heatmap.

S10

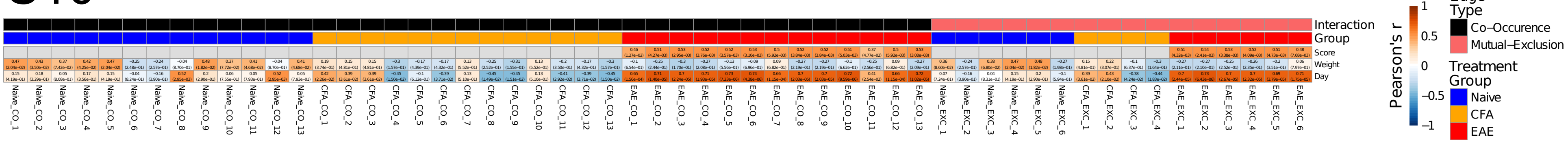
Interaction
Group
Score
Weight
Day
Naive_CO_1
Naive_CO_2
Naive_CO_3
Naive_CO_4
Naive_CO_5
Naive_CO_6
Naive_CO_7
Naive_CO_8
Naive_CO_9
Naive_CO_10
Naive_CO_11
Naive_CO_12
Naive_CO_13
CFA_CO_1
CFA_CO_2
CFA_CO_3
CFA_CO_4
CFA_CO_5
CFA_CO_6
CFA_CO_7
CFA_CO_8
CFA_CO_9
CFA_CO_10
CFA_CO_11
CFA_CO_12
CFA_CO_13
EAE_CO_1
EAE_CO_2
EAE_CO_3
EAE_CO_4
EAE_CO_5
EAE_CO_6
EAE_CO_7
EAE_CO_8
EAE_CO_9
EAE_CO_10
EAE_CO_11
EAE_CO_12
EAE_CO_13
Naive_EXC_1
Naive_EXC_2
Naive_EXC_3
Naive_EXC_4
Naive_EXC_5
Naive_EXC_6
CFA_EXC_1
CFA_EXC_2
CFA_EXC_3
CFA_EXC_4
EAE_EXC_1
EAE_EXC_2
EAE_EXC_3
EAE_EXC_4
EAE_EXC_5
EAE_EXC_6
Pearson's r
1
0.5
0
−0.5
−1
Edge Type
Co–Occurence
Mutual–Exclusion
Treatment Group
Naive
CFA
EAE

Figure S10: Heatmap of correlation coefficients between the primary principal components of each module and the observed body weights, dpi, and clinical scores in corresponding samples. Principal components were taken only from the samples and ASVs contained in each network module. Cells contain Pearson's r followed by a P-value in parentheses. Horizontal color bars represent network treatment identity (naïve, CFA, or EAE) and network type (co-occurrence or mutual exclusion).

S11

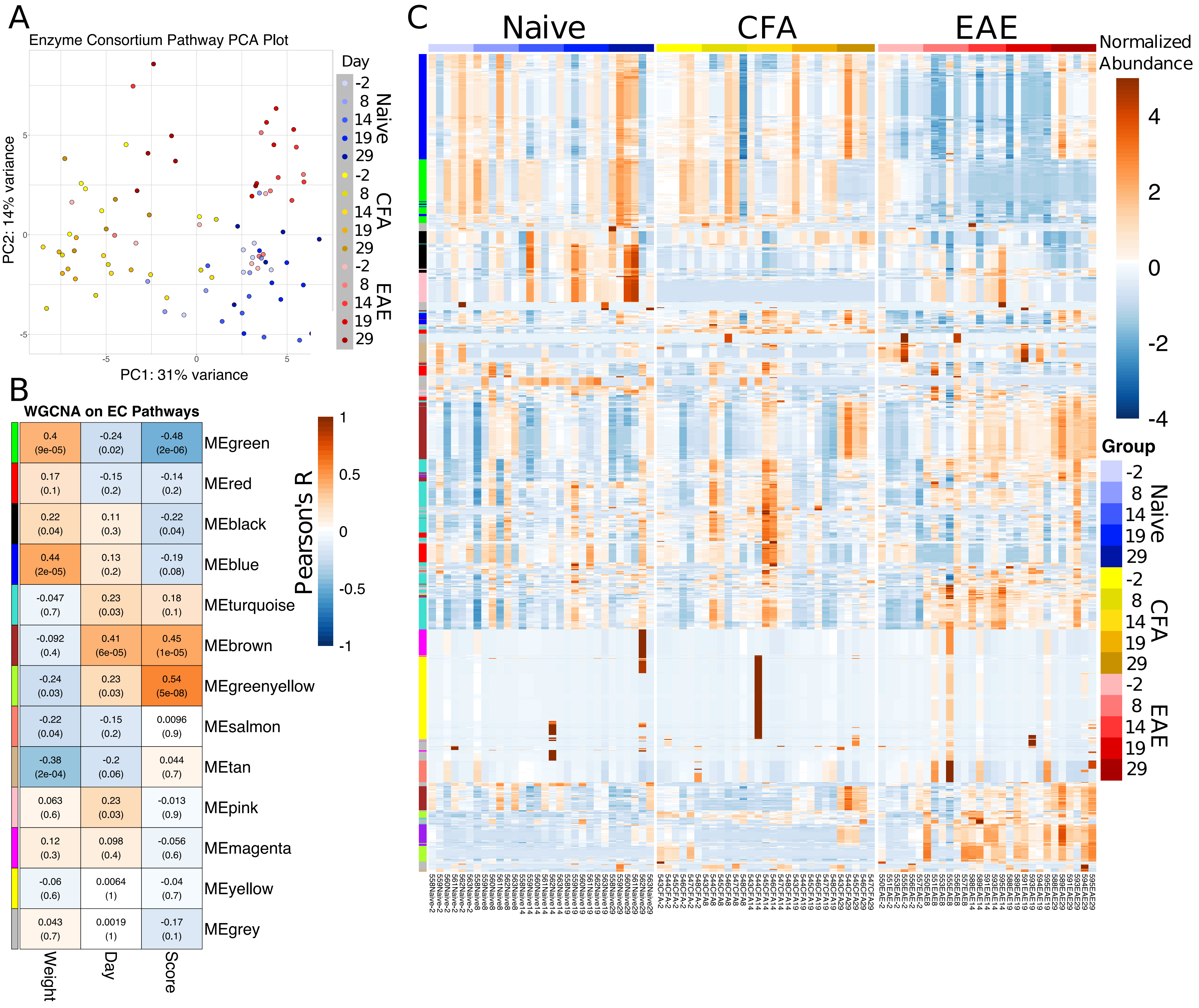

A
Enzyme Consortium Pathway PCA Plot
PC1: 31% variance
PC2: 14% variance
Day
-2
8
14
19
29
Naive
CFA
EAE
B
WGCNA on EC Pathways
0.4 (9e-05) | -0.24 (0.02) | -0.48 (2e-06) MEgreen
0.17 (0.1) | -0.15 (0.2) | -0.14 (0.2) MEred
0.22 (0.04) | 0.11 (0.3) | -0.22 (0.04) MEblack
0.44 (2e-05) | 0.13 (0.2) | -0.19 (0.08) MEblue
-0.047 (0.7) | 0.23 (0.03) | 0.18 (0.1) MEturquoise
-0.092 (0.4) | 0.41 (6e-05) | 0.45 (1e-05) MEbrown
-0.24 (0.03) | 0.23 (0.03) | 0.54 (5e-08) MEgreenyellow
-0.22 (0.04) | -0.15 (0.2) | 0.0096 (0.9) MEsalmon
-0.38 (2e-04) | -0.2 (0.06) | 0.044 (0.7) MEtan
0.063 (0.6) | 0.23 (0.03) | -0.013 (0.9) MEpink
0.12 (0.3) | 0.098 (0.4) | -0.056 (0.6) MEmagenta
-0.06 (0.6) | 0.0064 (1) | -0.04 (0.7) MEyellow
0.043 (0.7) | 0.0019 (1) | -0.17 (0.1) MEgrey
Weight
Day
Score
Pearson's R
1
0.5
0
-0.5
-1
C
Naive
CFA
EAE
Normalized Abundance
4
2
0
-2
-4
Group

Figure S11: Supplemental summary of WGCNA and subsequent analysis performed on EC accessions. **A**: PCA plot of first two principal components of EC accession data. Raw output from PICRUST2 was normalized and subject to a variance-stabilizing transformation within DESeq2. Samples diverge as dpi increases. **B**: Heatmap of modules derived from WGCNA on EC accessions. Cell color represents Pearson's r between the first principal component of each module and clinical score, dpi, and body weight. Vertical color bar represents the color for each WGCNA module. Cells contain the rounded Pearson's r and P-values in parentheses. **C**: Heatmap of all 1449 EC accessions with imputed abundances from PICRUSt2. Counts were first normalized with DESeq2 and then Z-score normalized for the final heatmap. Vertical color bar represents module membership and corresponds to modules listed in S10B. Horizontal bar represents the treatment and dpi of each sample.

S12

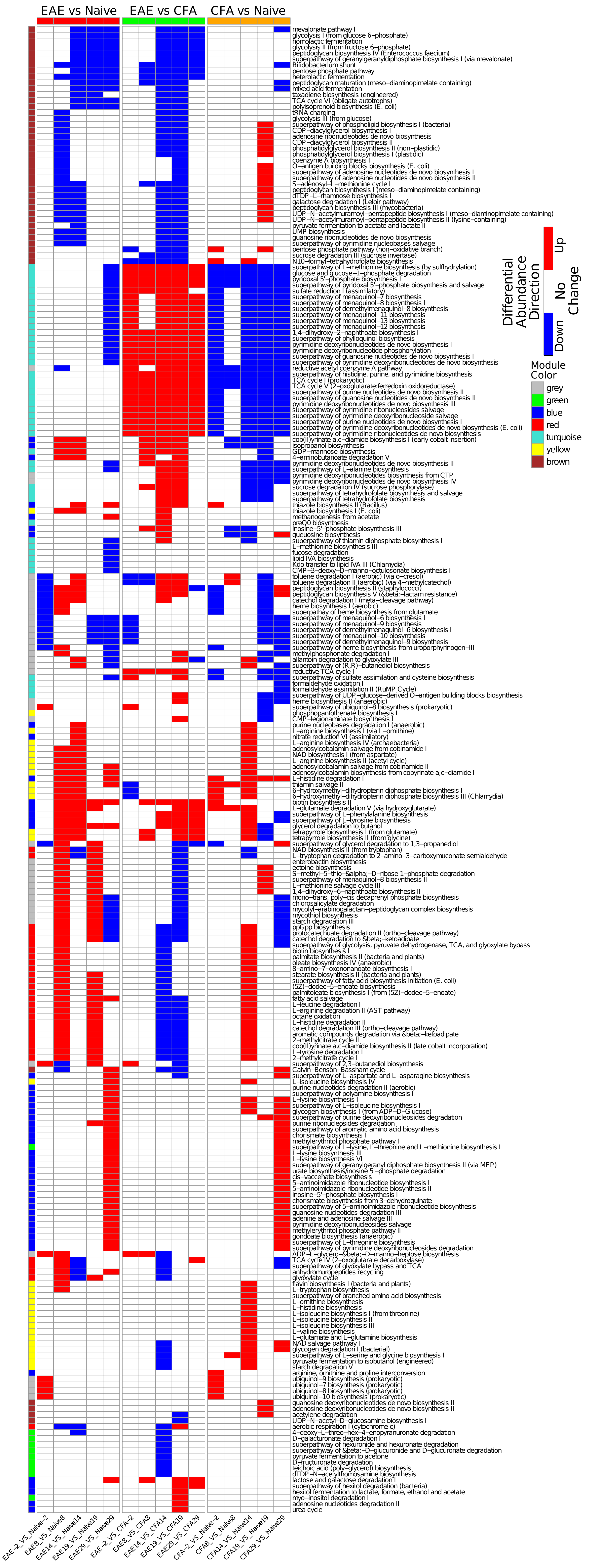

EAE vs Naive
EAE vs CFA
CFA vs Naive
mevalonate pathway I
glycolysis I (from glucose 6−phosphate)
homolactic fermentation
glycolysis II (from fructose 6−phosphate)
peptidoglycan biosynthesis IV (Enterococcus faecium)
superpathway of geranylgeranyldiphosphate biosynthesis I (via mevalonate)
Bifidobacterium shunt
pentose phosphate pathway
heterolactic fermentation
peptidoglycan maturation (meso−diaminopimelate containing)
mixed acid fermentation
taxadiene biosynthesis (engineered)
TCA cycle VI (obligate autotrophs)
polyisoprenoid biosynthesis (E. coli)
tRNA charging
glycolysis III (from glucose)
superpathway of phospholipid biosynthesis I (bacteria)
CDP−diacylglycerol biosynthesis I
adenosine ribonucleotides de novo biosynthesis
CDP−diacylglycerol biosynthesis II
phosphatidylglycerol biosynthesis II (non−plastidic)
phosphatidylglycerol biosynthesis I (plastidic)
coenzyme A biosynthesis I
O−antigen building blocks biosynthesis (E. coli)
superpathway of adenosine nucleotides de novo biosynthesis I
superpathway of adenosine nucleotides de novo biosynthesis II
S−adenosyl−L−methionine cycle I
peptidoglycan biosynthesis I (meso−diaminopimelate containing)
dTDP−L−rhamnose biosynthesis I
galactose degradation I (Leloir pathway)
peptidoglycan biosynthesis III (mycobacteria)
UDP−N−acetylmuramoyl−pentapeptide biosynthesis I (meso−diaminopimelate containing)
UDP−N−acetylmuramoyl−pentapeptide biosynthesis II (lysine−containing)
pyruvate fermentation to acetate and lactate II
UMP biosynthesis
guanosine ribonucleotides de novo biosynthesis
superpathway of pyrimidine nucleobases salvage
pentose phosphate pathway (non−oxidative branch)
sucrose degradation III (sucrose invertase)
N10−formyl−tetrahydrofolate biosynthesis
superpathway of L−methionine biosynthesis (by sulfhydrylation)
glucose and glucose−1−phosphate degradation
pyridoxal 5'−phosphate biosynthesis I
superpathway of pyridoxal 5'−phosphate biosynthesis and salvage
sulfate reduction I (assimilatory)
superpathway of menaquinol−7 biosynthesis
superpathway of menaquinol−8 biosynthesis I
superpathway of demethylmenaquinol−8 biosynthesis
superpathway of menaquinol−11 biosynthesis
superpathway of menaquinol−13 biosynthesis
superpathway of menaquinol−12 biosynthesis
1,4−dihydroxy−2−naphthoate biosynthesis I
superpathway of phylloquinol biosynthesis
pyrimidine deoxyribonucleotides de novo biosynthesis I
pyrimidine deoxyribonucleotide phosphorylation
superpathway of guanosine nucleotides de novo biosynthesis I
superpathway of pyrimidine deoxyribonucleotides de novo biosynthesis
reductive acetyl coenzyme A pathway
superpathway of histidine, purine, and pyrimidine biosynthesis
TCA cycle I (prokaryotic)
TCA cycle V (2−oxoglutarate:ferredoxin oxidoreductase)
superpathway of purine nucleotides de novo biosynthesis II
superpathway of guanosine nucleotides de novo biosynthesis II
pyrimidine deoxyribonucleotides de novo biosynthesis III
superpathway of pyrimidine ribonucleosides salvage
superpathway of pyrimidine deoxyribonucleoside salvage
superpathway of purine nucleotides de novo biosynthesis I
superpathway of pyrimidine deoxyribonucleotides de novo biosynthesis (E. coli)
superpathway of pyrimidine ribonucleotides de novo biosynthesis
cob(II)yrinate a,c−diamide biosynthesis I (early cobalt insertion)
isopropanol biosynthesis
GDP−mannose biosynthesis
4−aminobutanoate degradation V
pyrimidine deoxyribonucleotides de novo biosynthesis II
superpathway of L−alanine biosynthesis
pyrimidine deoxyribonucleotides biosynthesis from CTP
pyrimidine deoxyribonucleotides de novo biosynthesis IV
sucrose degradation IV (sucrose phosphorylase)
superpathway of tetrahydrofolate biosynthesis and salvage
superpathway of tetrahydrofolate biosynthesis
thiazole biosynthesis II (Bacillus)
thiazole biosynthesis I (E. coli)
methanogenesis from acetate
preQ0 biosynthesis
inosine−5'−phosphate biosynthesis III
queuosine biosynthesis
superpathway of thiamin diphosphate biosynthesis I
L−methionine biosynthesis III
fucose degradation
lipid IVA biosynthesis
Kdo transfer to lipid IVA III (Chlamydia)
CMP−3−deoxy−D−manno−octulosonate biosynthesis I
toluene degradation I (aerobic) (via o−cresol)
toluene degradation II (aerobic) (via 4−methylcatechol)
peptidoglycan biosynthesis II (staphylococci)
peptidoglycan biosynthesis V (β−lactam resistance)
catechol degradation I (meta−cleavage pathway)
heme biosynthesis I (aerobic)
superpathay of heme biosynthesis from glutamate
superpathway of menaquinol−6 biosynthesis I
superpathway of menaquinol−9 biosynthesis
superpathway of demethylmenaquinol−6 biosynthesis I
superpathway of menaquinol−10 biosynthesis
superpathway of demethylmenaquinol−9 biosynthesis
superpathway of heme biosynthesis from uroporphyrinogen−III
methylphosphonate degradation I
allantoin degradation to glyoxylate III
superpathway of (R,R)−butanediol biosynthesis
reductive TCA cycle I
superpathway of sulfate assimilation and cysteine biosynthesis
formaldehyde oxidation I
formaldehyde assimilation II (RuMP Cycle)
superpathway of UDP−glucose−derived O−antigen building blocks biosynthesis
heme biosynthesis II (anaerobic)
superpathway of ubiquinol−8 biosynthesis (prokaryotic)
phosphopantothenate biosynthesis I
CMP−legionaminate biosynthesis I
purine nucleobases degradation I (anaerobic)
L−arginine biosynthesis I (via L−ornithine)
nitrate reduction VI (assimilatory)
L−arginine biosynthesis IV (archaebacteria)
adenosylcobalamin salvage from cobinamide I
NAD biosynthesis I (from aspartate)
L−arginine biosynthesis II (acetyl cycle)
adenosylcobalamin salvage from cobinamide II
adenosylcobalamin biosynthesis from cobyrinate a,c−diamide I
L−histidine degradation I
thiamin salvage II
6−hydroxymethyl−dihydropterin diphosphate biosynthesis I
6−hydroxymethyl−dihydropterin diphosphate biosynthesis III (Chlamydia)
biotin biosynthesis II
L−glutamate degradation V (via hydroxyglutarate)
superpathway of L−phenylalanine biosynthesis
superpathway of L−tyrosine biosynthesis
glycerol degradation to butanol
tetrapyrrole biosynthesis I (from glutamate)
tetrapyrrole biosynthesis II (from glycine)
superpathway of glycerol degradation to 1,3−propanediol
NAD biosynthesis II (from tryptophan)
L−tryptophan degradation to 2−amino−3−carboxymuconate semialdehyde
enterobactin biosynthesis
ectoine biosynthesis
S−methyl−5−thio−α−D−ribose 1−phosphate degradation
superpathway of menaquinol−8 biosynthesis II
L−methionine salvage cycle III
1,4−dihydroxy−6−naphthoate biosynthesis II
mono−trans, poly−cis decaprenyl phosphate biosynthesis
chlorosalicylate degradation
mycolyl−arabinogalactan−peptidoglycan complex biosynthesis
mycothiol biosynthesis
starch degradation III
ppGpp biosynthesis
protocatechuate degradation II (ortho−cleavage pathway)
catechol degradation to β−ketoadipate
superpathway of glycolysis, pyruvate dehydrogenase, TCA, and glyoxylate bypass
biotin biosynthesis I
palmitate biosynthesis II (bacteria and plants)
oleate biosynthesis IV (anaerobic)
8−amino−7−oxononanoate biosynthesis I
stearate biosynthesis II (bacteria and plants)
superpathway of fatty acid biosynthesis initiation (E. coli)
(5Z)−dodec−5−enoate biosynthesis
palmitoleate biosynthesis I (from (5Z)−dodec−5−enoate)
fatty acid salvage
L−leucine degradation I
L−arginine degradation II (AST pathway)
octane oxidation
L−histidine degradation II
catechol degradation III (ortho−cleavage pathway)
aromatic compounds degradation via β−ketoadipate
2−methylcitrate cycle II
cob(II)yrinate a,c−diamide biosynthesis II (late cobalt incorporation)
L−tyrosine degradation I
2−methylcitrate cycle I
superpathway of 2,3−butanediol biosynthesis
Calvin−Benson−Bassham cycle
superpathway of L−aspartate and L−asparagine biosynthesis
L−isoleucine biosynthesis IV
purine nucleotides degradation II (aerobic)
superpathway of polyamine biosynthesis I
L−lysine biosynthesis I
superpathway of L−isoleucine biosynthesis I
glycogen biosynthesis I (from ADP−D−Glucose)
superpathway of purine deoxyribonucleosides degradation
purine ribonucleosides degradation
superpathway of aromatic amino acid biosynthesis
chorismate biosynthesis I
methylerythritol phosphate pathway I
superpathway of L−lysine, L−threonine and L−methionine biosynthesis I
L−lysine biosynthesis III
L−lysine biosynthesis VI
superpathway of geranylgeranyl diphosphate biosynthesis II (via MEP)
urate biosynthesis/inosine 5'−phosphate degradation
cis−vaccenate biosynthesis
5−aminoimidazole ribonucleotide biosynthesis I
5−aminoimidazole ribonucleotide biosynthesis II
inosine−5'−phosphate biosynthesis I
chorismate biosynthesis from 3−dehydroquinate
superpathway of 5−aminoimidazole ribonucleotide biosynthesis
guanosine nucleotides degradation III
adenine and adenosine salvage III
pyrimidine deoxyribonucleosides salvage
methylerythritol phosphate pathway II
gondoate biosynthesis (anaerobic)
superpathway of L−threonine biosynthesis
superpathway of pyrimidine deoxyribonucleosides degradation
ADP−L−glycero−β−D−manno−heptose biosynthesis
TCA cycle IV (2−oxoglutarate decarboxylase)
superpathway of glyoxylate bypass and TCA
anhydromuropeptides recycling
glyoxylate cycle
flavin biosynthesis I (bacteria and plants)
L−tryptophan biosynthesis
superpathway of branched amino acid biosynthesis
L−ornithine biosynthesis
L−histidine biosynthesis
L−isoleucine biosynthesis I (from threonine)
L−isoleucine biosynthesis II
L−isoleucine biosynthesis III
L−valine biosynthesis
L−glutamate and L−glutamine biosynthesis
NAD salvage pathway I
glycogen degradation I (bacterial)
superpathway of L−serine and glycine biosynthesis I
pyruvate fermentation to isobutanol (engineered)
starch degradation V
arginine, ornithine and proline interconversion
ubiquinol−9 biosynthesis (prokaryotic)
ubiquinol−7 biosynthesis (prokaryotic)
ubiquinol−8 biosynthesis (prokaryotic)
ubiquinol−10 biosynthesis (prokaryotic)
guanosine deoxyribonucleotides de novo biosynthesis II
adenosine deoxyribonucleotides de novo biosynthesis II
acetylene degradation
UDP−N−acetyl−D−glucosamine biosynthesis I
aerobic respiration I (cytochrome c)
4−deoxy−L−threo−hex−4−enopyranuronate degradation
D−galacturonate degradation I
superpathway of hexuronide and hexuronate degradation
superpathway of β−D−glucuronide and D−glucuronate degradation
pyruvate fermentation to acetone
D−fructuronate degradation
teichoic acid (poly−glycerol) biosynthesis
dTDP−N−acetylthomosamine biosynthesis
lactose and galactose degradation I
superpathway of hexitol degradation (bacteria)
hexitol fermentation to lactate, formate, ethanol and acetate
myo−inositol degradation I
adenosine nucleotides degradation II
urea cycle
EAE−2_VS_Naive−2
EAE8_VS_Naive8
EAE14_VS_Naive14
EAE19_VS_Naive19
EAE29_VS_Naive29
EAE−2_VS_CFA−2
EAE8_VS_CFA8
EAE14_VS_CFA14
EAE19_VS_CFA19
EAE29_VS_CFA29
CFA−2_VS_Naive−2
CFA8_VS_Naive8
CFA14_VS_Naive14
CFA19_VS_Naive19
CFA29_VS_Naive29
Differential Abundance Direction
Up
No Change
Down
Module Color
grey
green
blue
red
turquoise
yellow
brown

Figure S12: Unabridged heatmap created using same procedures as the heatmap of EC pathway differential abundance analysis outcomes in Fig. 5D. Cell color represents the directionality and significance of the log2 fold change vales for each pairwise comparison. Red represents significant increase ($P<=0.05$, $L2FC>0$), blue represents significant decrease ($P<=0.05$, $L2FC<0$), and white represents no significant change ($P>0.05$). Each row represents an EC pathway of interest and column numbering represents the dpi at which a pairwise comparison was made. Vertical color bar denotes the module membership of each EC pathway.

S13

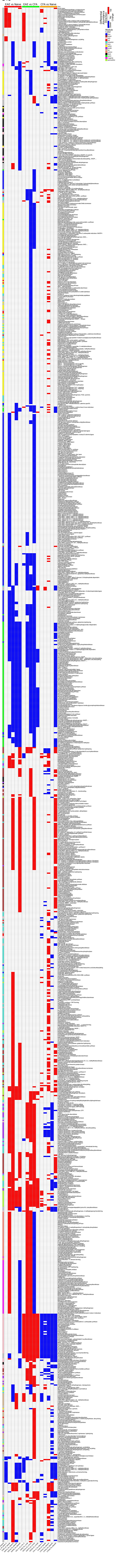
EAE vs Naive
EAE vs CFA
CFA vs Naive
Test
Differential Abundance Direction
Up
No Change
Down
Module Color
blue
brown
pink
red
turquoise
grey
yellow
purple
tan
salmon
black
green
magenta
greenyellow

Figure S13: Unabridged heatmap created using same procedures as the heatmap of EC pathway differential abundance analysis outcomes in Fig. 5D. Heatmap representing differential abundance analysis results for EC accessions. Cell color represents the directionality and significance of the log2 fold change vales for each pairwise comparison. Red represents significant increase ($P<=0.05$, $L2FC>0$), blue represents significant decrease ($P<=0.05$, $L2FC<0$), and white represents no significant change ($P>0.05$). Each row represents an EC accession of interest and column numbering represents the dpi at which a pairwise comparison was made. Vertical color bar denotes the module membership of each EC accession.
